# Prevalence of Hepatocellular Carcinoma in Hepatitis B Population within Southeast Asia: A Systematic Review and Meta-Analysis of 39,050 Participants

**DOI:** 10.3390/pathogens12101220

**Published:** 2023-10-06

**Authors:** Ali A. Rabaan, Kizito Eneye Bello, Ahmad Adebayo Irekeola, Nawal A. Al Kaabi, Muhammad A. Halwani, Amjad A. Yousuf, Amer Alshengeti, Amal H. Alfaraj, Faryal Khamis, Maha F. Al-Subaie, Bashayer M. AlShehail, Souad A. Almuthree, Noha Y. Ibraheem, Mahassen H. Khalifa, Mubarak Alfaresi, Mona A. Al Fares, Mohammed Garout, Ahmed Alsayyah, Ahmad A. Alshehri, Ali S. Alqahtani, Mohammed Alissa

**Affiliations:** 1 Molecular Diagnostic Laboratory, Johns Hopkins Aramco Healthcare, Dhahran 31311, Saudi Arabia; 2 College of Medicine, Alfaisal University, Riyadh 11533, Saudi Arabia; 3 Department of Public Health and Nutrition, The University of Haripur, Haripur 22610, Pakistan; 4 Department of Microbiology, Faculty of Natural Science, Kogi State University (Prince Abubakar Audu University) Anyigba, Anyigba PMB 1008, Nigeria; 5 Department of Medical Microbiology and Parasitology, School of Medical Sciences, Health Campus, Universiti Sains Malaysia, Kubang Kerian 16150, Kelantan, Malaysia; 6 Microbiology Unit, Department of Biological Sciences, College of Natural and Applied Sciences, Summit University Offa, Offa PMB 4412, Nigeria; 7 College of Medicine and Health Science, Khalifa University, Abu Dhabi 127788, United Arab Emirates; 8 Sheikh Khalifa Medical City, Abu Dhabi Health Services Company (SEHA), Abu Dhabi 51900, United Arab Emirates; 9 Department of Medical Microbiology, Faculty of Medicine, Al Baha University, Al Baha 4781, Saudi Arabia; 10 Department of Medical Laboratories Technology, College of Applied Medical Sciences, Taibah University, Madinah 41411, Saudi Arabia; 11 Department of Pediatrics, College of Medicine, Taibah University, Al-Madinah 41491, Saudi Arabia; 12 Department of Infection Prevention and Control, Prince Mohammad Bin Abdulaziz Hospital, National Guard Health Affairs, Al-Madinah 41491, Saudi Arabia; 13 Pediatric Department, Abqaiq General Hospital, First Eastern Health Cluster, Abqaiq 33261, Saudi Arabia; 14 Infection Diseases Unit, Department of Internal Medicine, Royal Hospital, Muscat 1331, Oman; 15 Research Center, Dr. Sulaiman Alhabib Medical Group, Riyadh 13328, Saudi Arabia; 16 Department of Infectious Diseases, Dr. Sulaiman Alhabib Medical Group, Riyadh 13328, Saudi Arabia; 17 Pharmacy Practice Department, College of Clinical Pharmacy, Imam Abdulrahman Bin Faisal University, Dammam 31441, Saudi Arabia; 18 Department of Infectious Disease, King Abdullah Medical City, Makkah 43442, Saudi Arabia; 19 Department of Pathology and Laboratory Medicine, Zayed Military Hospital, Abu Dhabi 3740, United Arab Emirates; 20 Department of Pathology, College of Medicine, Mohammed Bin Rashid University of Medicine and Health Sciences, Dubai 505055, United Arab Emirates; 21 Department of Internal Medicine, King Abdulaziz University Hospital, Jeddah 21589, Saudi Arabia; 22 Department of Community Medicine and Health Care for Pilgrims, Faculty of Medicine, Umm Al-Qura University, Makkah 21955, Saudi Arabia; 23 Department of Pathology, College of Medicine, Imam Abdulrahman Bin Faisal University, Dammam 31441, Saudi Arabia; 24 Department of Clinical Laboratory Sciences, Faculty of Applied Medical Sciences, Najran University, Najran 61441, Saudi Arabia; 25 Department of Medical Laboratory Sciences, Faculty of Applied Medical Sciences, King Khalid University, Abha 61481, Saudi Arabia; 26 Department of Medical Laboratory Sciences, College of Applied Medical Sciences, Prince Sattam bin Abdulaziz University, Al-Kharj 11942, Saudi Arabia

**Keywords:** hepatocellular carcinoma, hepatitis B virus, liver disease, Southeast Asia, prevalence, systematic review, meta-analysis

## Abstract

Background and aim: Hepatocellular carcinoma (HCC) is a significant complication of hepatitis B and still poses a global public health concern. This systematic review and meta-analysis provide adequate details on the prevalence of HCC in the HBV population within Southeast Asian countries. Method: Following the Preferred Reporting Items for Systematic Reviews and Meta-analysis (PRISMA) criteria, a thorough search for literature discussing the prevalence of HCC in the HBV population within southeast Asia was performed. Eligible studies were subjected to a meta-analysis utilising a DerSimonian and Laird approach and a random effect model. A protocol was registered with PROSPERO (CRD42023423953). Result: Our study meticulously recovered 41 articles from seven countries in Southeast Asia, namely Cambodia, Indonesia, Malaysia, the Philippines, Singapore, Thailand, and Vietnam. A total of 39,050 HBV patients and 7479 HCC cases in southeast Asia were analysed. The pooled prevalence of HCC in HBV cases within southeast Asia was 45.8% (95% CI, 34.3–57.8%, *I*^2^ = 99.51%, *p* < 0.001). Singapore (62.5%, CI: 42.4–79.1) had the highest pooled prevalence of HCC in the HBV population compared to Vietnam, with the lowest estimate (22.4%, CI: 9.9–44.9). There was a drop in the pooled prevalence of HCC in HBV from 2016 until now (37.6%, CI: 19.2–60.5). Conclusion: The findings of this review reveal a high pooled prevalence of HCC in the HBV population and therefore stir the need for routine screening, management, and surveillance.

## 1. Introduction

The most typical primary liver cancer, hepatocellular carcinoma (HCC), presents a severe health problem worldwide [1]. The prevalence of HCC among HBV patients in Southeast Asia, where chronic hepatitis B virus (HBV) infection is still common, is of significant concern [2,3]. The World Health Organisation estimates that almost 100 million people in the region have chronic HBV infections. Being chronically infected with HBV considerably raises the likelihood of developing HCC, posing a daunting obstacle for local healthcare systems [4]. 

Southeast Asia exhibits distinctive epidemiological patterns regarding the frequency of HCC in HBV patients. This variation is influenced by several variables, such as virus genotypes, host immune responses, environmental conditions, and dietary habits [5,6,7]. Designing efficient prevention and control methods suited to the region’s demands requires understanding the complex nature of HCC prevalence and unravelling the underlying mechanisms [4]. According to studies, having a chronic HBV infection increases the likelihood of developing HCC [2,3,8,9]. The intricate interaction between viral replication, the host immune system, and a person’s genetic makeup is a significant factor in predicting the risk that HCC would develop in an HBV patient [10,11]. 

In chronic and asymptomatic cases, the presence of the infection might go unnoticed over the span of years, even as the liver continues to produce significant amounts of viral antigens and particles [12]. Despite this, the immune response triggered by HBV after many years of infection is often insufficient to eliminate all infected liver cells. This leads to persistent inflammation and progressive damage to the liver [13,14]. Consequently, a repetitive cycle of liver damage and regeneration ensues, ultimately fostering the development of tumours [12].

HBV employs various mechanisms to facilitate tumor formation, primarily by influencing different pathways that either activate or deactivate specific processes, thus contributing to the development of HCC [12]. Notably, HBV is distinct among hepatotropic viruses due to its ability to induce HCC without the presence of cirrhosis. Nonetheless, a significant majority of HBV-related HCC cases occur in patients with cirrhosis [15]. The culmination of HBV-related liver disease progression is liver cirrhosis, which undoubtedly stands as the primary risk factor for the emergence of HCC. Immunological markers play a pivotal role in the process of HBV-related HCC oncogenesis [16].

Numerous stages of the viral and hepatocyte life cycles are directly and indirectly implicated, along with the disruption of microenvironmental balance [17]. Mutations within the HBV genome are linked to an elevated risk of HCC, potentially affecting any of the HBV genes [18]. Furthermore, the distribution of HBV genotypes also influences the progression of HBV-related HCC. Genotypes B and C of HBV have a higher rate of progression to HCC compared to other genotypes [19].

Although early diagnosis of HCC is essential for effective intervention, missed early signs frequently result in delayed diagnosis. The various genomic traits of HCC in HBV patients also make treatment selection challenging, highlighting the necessity for precision medicine strategies to attain the best results [20,21,22]. Despite the difficulties, there have been notable advancements in the fight against HCC in HBV patients in various Southeast Asian nations [2,23]. For the early detection and better management of HCC cases, improvements in diagnostic methods, targeted medicines, and surveillance programme developments show promise. Additionally, continuing studies into cutting-edge therapy modalities, including immunotherapies and gene treatments, may pave the way for future treatment choices that are more successful [20,24,25,26]. 

## 2. Materials and Methods

### 2.1. Search for Studies

We started a preliminary key term search on two review databases, PROSPERO and DARE, to ensure the thorough synthesis of this review without repetition or redundancy of current information or ongoing projects. A protocol was created for this study and registered on PROSPERO (ID: CRD42023423953). We conducted a comprehensive search across four well-known international electronic databases, namely PubMed, Scopus, Science Direct, and Google Scholar, adhering to the Preferred Reporting Items for Systematic Reviews and Meta-analysis standards for robust synthesis [27]. We were looking for publications on the prevalence of HBV and its co-infection with hepatocellular cancer in Asian nations. We used various search techniques, including critical terms like “hepatitis B” and “hepatocellular carcinoma” and an extensive list of Southeast Asian countries. We used abbreviations like “HBV” and “HCC”, synonymous keyword variations like “liver cancer”, and Boolean operators as appropriate to broaden the scope of our search. We did not limit the search to language and publication year to ensure the validity of our findings. In Supplementary File S1, information on the search strategies used across the four electronic databases is detailed. The last search was carried out on 23 February 2023. All the search results retrieved from the various databases were painstakingly incorporated into the Mendeley desktop reference manager programme for the removal of duplicate records and screening.

### 2.2. Eligibility and Data Extraction 

Cross-sectional studies, prospective cohorts, and retrospective cohorts with HBV and HCC data obtained from Southeast Asian nations were all included in this review after a careful procedure of inclusion and exclusion. We excluded review papers, editorials, case reports, short communications, conference proceedings, and articles without well-specified sample sources and origins. In addition, we did not include studies whose whole text could not be recovered or whose data were redundant or duplicated. 

Three authors (KEB, AAI, and AAA) independently reviewed each title, abstract, and full-text submission based on the inclusion criteria. In cases of disagreement during the review, discussion among the authors was employed to reach a consensus. The qualified studies’ full texts, abstracts, and titles under the relevant headings were thoroughly read. Relevant information, including the authors’ names, the year the work was published, the nation of study, the study designs, gender, HBV genotypes, presence of cirrhosis, treatment status, stages of HCC disease, and presence of a tumour marker (alpha-fetoprotein), was extracted into a structured data extraction sheet. The data was painstakingly retrieved by the authors.

### 2.3. Statistical Analysis and Quality Assessment

We used the random-effects model and the DerSimonian and Laird meta-analysis approaches in our research to ascertain the pooled prevalence of HCC in HBV patients. OpenMeta and Comprehensive Meta-analysis Software were used for analysis [28]. We used a funnel plot to measure the bias in publication. Cochran’s Q test was used to assess the heterogeneity of subgroup estimates. Statistically, the Cochran Q test and I^2^ values were used to calculate the heterogeneity index, with I^2^ values of 25%, 50%, and 75%, respectively, denoting low, moderate, and high levels of heterogeneity [29,30]. 

We did a subgroup analysis to assess the prevalence of HCC in HBV across various geographies, study kinds, years of publication, gender, stages of HCC, the presence of cirrhosis, and the presence of alpha-fetoprotein (AFP) to provide additional data. OpenMeta Analyst software was used to conduct this subgroup analysis [31]. A *p*-value of less than 0.001 was regarded as statistically significant in each test. We used the Joanna Briggs Institute (JBI) critical assessment checklist for prevalence statistics to ensure the overall quality of the included papers [32]. (Supplementary File S2). The authors carefully examined the studies and gave each study a score of “2” for “yes” and “0” for “no” to create a quality score that ranged from 0 to 18. Studies with a quality score between 14 and 18 were deemed sufficient. Supplementary Table S1 gives specifics on the 41 included studies’ quality evaluation.

## 3. Results

### 3.1. Search Results and Eligible Studies

**Figure 1:**
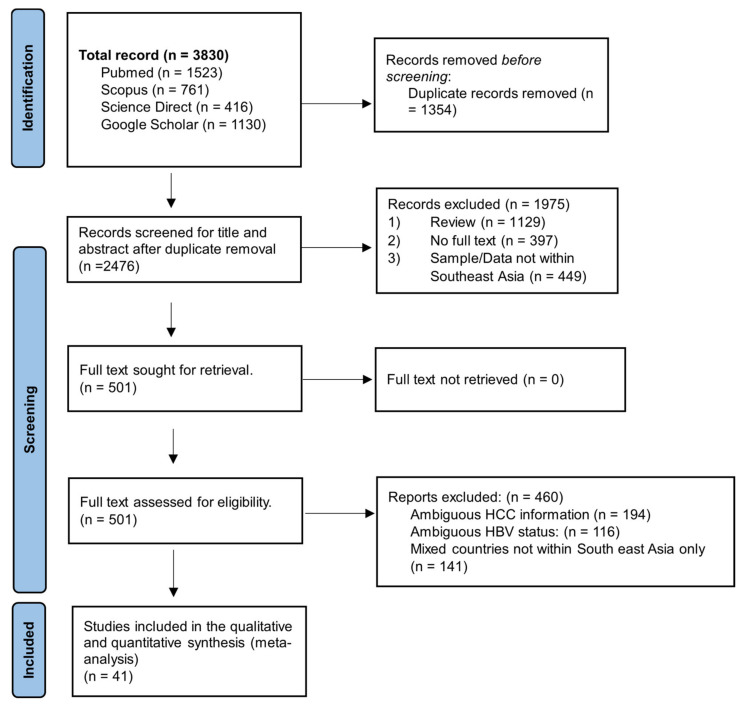
Flow chart showing an overview of the article selection process.

Our thorough search of four electronic databases yielded a total of 3830 articles. We carefully examined every record and removed duplicates to provide a well-curated selection of 2476 articles. After additional screening based on titles and abstracts, 1975 items were eliminated, leaving 501 papers for in-depth full-text review. However, 460 articles were disqualified during this evaluation stage because they did not adhere to the inclusion criteria. Figure 1 shows a thorough representation of the selection procedure. In the end, 41 publications were analysed, totalling 39,050 HBV patients and 7479 HCC cases in southeast Asia.

Our study meticulously recovered 41 articles from seven countries in Southeast Asia, namely Cambodia, Indonesia, Malaysia, the Philippines, Singapore, Thailand, and Vietnam. Thailand emerged as the primary contributor, accounting for 19 studies (*n* = 19). Within Southeast Asia, a total of 7479 cases of HCC were reported among 39,050 HBV cases, spanning diverse populations and clinical settings. Across the 41 studies, the number of HCC cases varied significantly, ranging from 2743 (Singapore) to 226 (Cambodia) (Table 1). There has been a notable concentration of articles published since 2016. The meta-analysis had three major study designs (case-control, cross-sectional, and retrospective). Cross-sectional study designs had the highest number of articles (*n* = 20) compared to case-control studies (*n* = 6).

The review revealed a broad age distribution, from as young as 1 to as old as 93. However, the male category had a higher pooled prevalence (60.3%). This disparity in gender representation resonates with the intricate diversities inherent in the field of study, as represented in Table 1. Furthermore, two studies reported that HBV genotypes and cirrhosis prevalence vary within the study. The stages of HCC identified in the included literature included early, intermediate, and late stages (Table 1). Early cases were defined as cases with a singular tumour with a tumour size less than 2 cm and not in the blood stream; intermediate cases were defined as cases with multiple tumours just progressing into the blood stream. Tertiary cases were defined as multiple tumours with evidence of bloodstream circulation.

**Figure 2:**
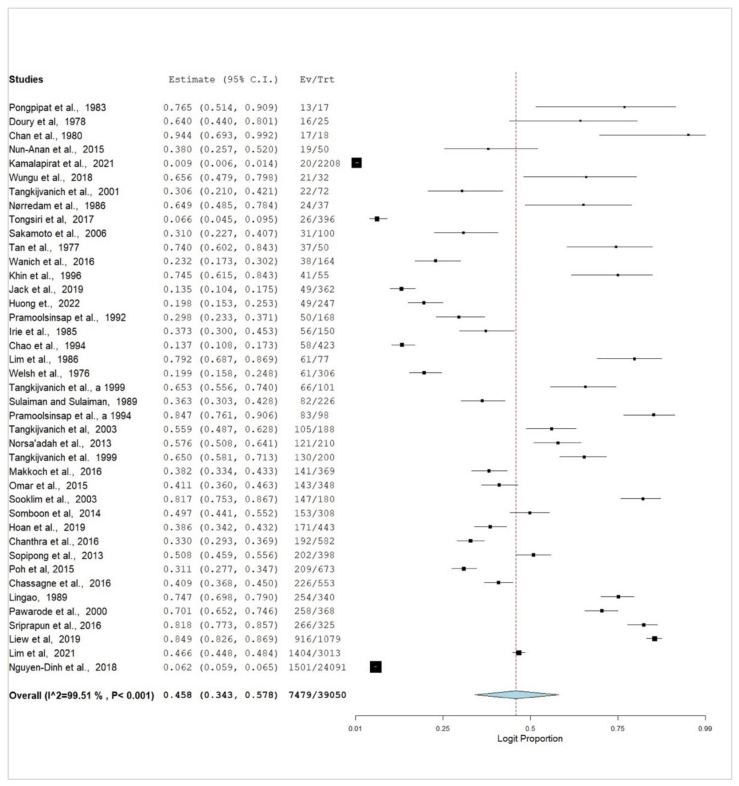
Forest plot for the pooled prevalence of HCC in HBV within Southeast Asia [5,6,8,33,34,35,36,37,38,39,40,41,42,43,44,45,46,47,48,49,50,51,52,53,54,55,56,57,58,59,60,61,62,63,64,65,66,67,68,69,70].

**Figure 3:**
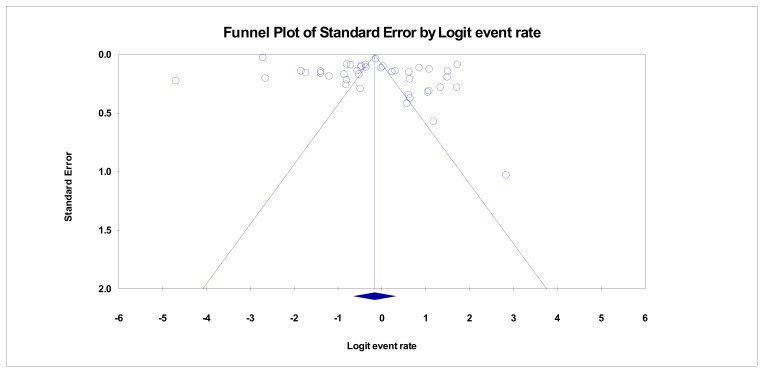
Funnel plot of HCC in HBV cases within Southeast Asia (Egger’s *p* = 0.00184).

There was a high prevalence of HCC in HBV subjects in Southeast Asia. The pooled prevalence of HCC in HBV cases was 45.8% (95% CI, 34.3–57.8%) (Figure 2). There was also high heterogeneity (*I*^2^ = 99.51%, *p* < 0.001). The funnel plot shows no evidence of significant publication bias in the pooled prevalence of HCC in HBV cases within southeast Asia (Figure 3).

### 3.2. Subgroup Meta-Analysis

**Figure 4:**
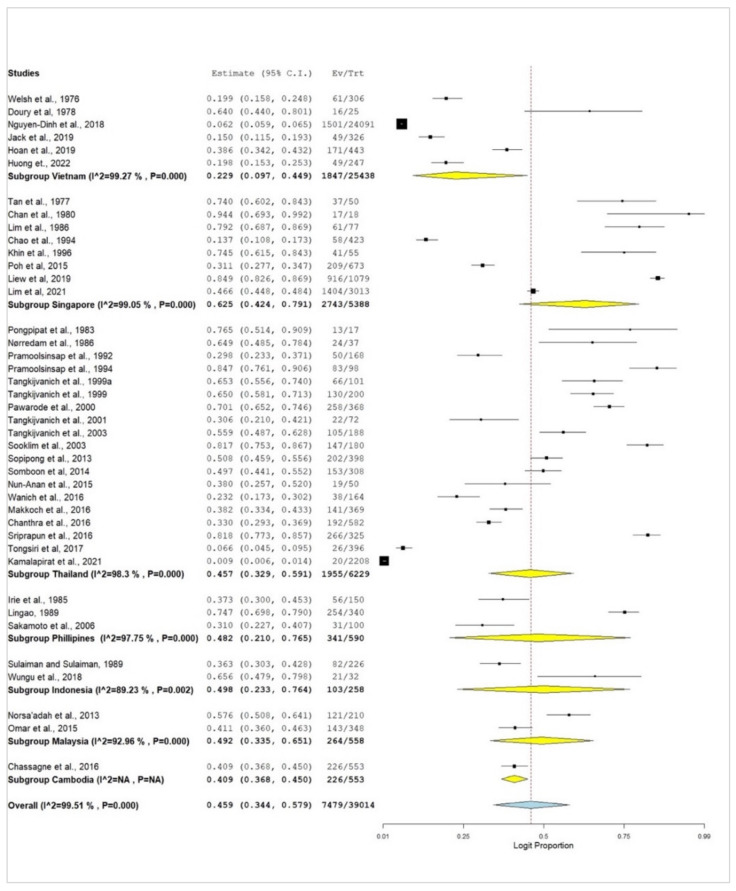
Subgroup forest plot of HCC in HBV within southeast Asia in relation to the country of study [5,6,8,33,34,35,36,37,38,39,40,41,42,43,44,45,46,47,48,49,50,51,52,53,54,55,56,57,58,59,60,61,62,63,64,65,66,67,68,69,70].

The subgroup meta-analysis reveals the pooled prevalence in relation to countries of study, type of study, year of publication, gender, HBV genotypes, presence of cirrhosis, stages of HCC, and presence of tumour markers. There was high diversity in the pooled prevalence of HCC in HBV cases within southeast Asia. Thailand had the highest number of studies (*n* = 19), but Singapore had the highest pooled prevalence (62.5% [95% CI: 42.4–79.1%, *I*^2^ = 99.05%, *p* < 0.001]) with only eight studies. Vietnam had the lowest prevalence (22.9%). The heterogeneity test did not apply to the study from Cambodia. Six out of seven countries (Vietnam, Singapore, Thailand, the Philippines, Indonesia, and Malaysia) had high levels of heterogeneity (*I*^2^ *≥* 90%), as represented in Figure 4 and Table 2.

A stark contrast was observed between males and females. The prevalence of the condition was notably higher in males (18.6% [95% CI: 14.3–23.9]). Females, on the other hand, exhibited a much lower prevalence of 6.9%. Based on the HBV genotypes reported, a similar prevalence of 0.3% was found for individuals with genotypes A, B, and C (Table 2). Furthermore, individuals with cirrhosis exhibit a prevalence of 6.7%. Conversely, the absence of cirrhosis yields a prevalence of 4.1% (Table 2).

Based on the stage of HCC, a pooled prevalence of 4.1% was found for subjects with early-stage HCC, 1.1% for those with intermediate, and 1.3% for those with late-stage disease (Table 2). Those with elevated AFP levels demonstrate a prevalence of 0.7% (95% CI: 0.5–1.0). In contrast, the absence of elevated AFP yields a prevalence of 0.6% (95% CI: 0.3–1.2%), as represented in Table 2.

Three types of study designs were included in this systematic review and meta-analysis (cross-sectional, retrospective, and case-control study designs). Cross-sectional studies had the highest number of studies (*n* = 21) compared to case-control studies, with the fewest included studies (*n* = 6). Cross-sectional studies had the highest pooled prevalence estimate (53.0% [95% CI: 39.1–66.5%, *I*^2^ = 97.86%, *p* < 0.001]). Regardless of the number of studies (*n* = 14), retrospective study designs had the lowest pooled prevalence in this category (36.5% (95% CI: 18.7–59.0%, *I*^2^ = 99.79%, *p* < 0.001)) but had the highest heterogeneity level. The corresponding forest plot is provided in File S4 of the Supplementary File. The year of publication for the included studies spans from 1976–2022 (46 years). The year group with the most publications was within the last decade (2016–now) (*n* = 14). Despite the high number of studies, this year’s category had the lowest pooled HCC in HBV prevalence estimate within Southeast Asia (37.6% [95% CI: 19.2–60.5%, *I*^2^ = 99.78%, *p* < 0.001]) in comparison to year group 1975–1985 with the highest pooled prevalence (58.0% (95% CI: 37.1–76.4%, *I*^2^ = 97.66%, *p* < 0.001)). The corresponding forest plot is shown in File S4 of the Supplementary File. 

## 4. Discussion

A systematic review and meta-analysis were performed to ascertain the pooled prevalence of HCC in HBV cases in Southeast Asia. The results of this study were based on the papers found in seven out of the eleven Southeast Asian nations (Cambodia, Indonesia, Malaysia, the Philippines, Singapore, Thailand, and Vietnam) that reported cases of HCC in HBV. We could locate 41 articles through our search strategy, making up a varied and representative collection from this area. Thailand emerged as the primary contributor to the literature corpus among the nations examined, exhibiting a great quantity of 19 studies. A total of 39,050 instances of the hepatitis B virus (HBV) were reported across the included studies, constituting a sizable sample size for this substantial body of research. A startling 7479 instances of hepatocellular carcinoma (HCC) were recorded within this cohort, offering important information about the prevalence and clinical characteristics of HCC in HBV cohorts within Southeast Asia [2,3,9,71]. 

The heterogeneity in the distribution of HCC cases among the 41 studies highlights the variety of demographics and clinical settings under study. Table 1 shows that Singapore had the largest number of HCC cases (2743 cases), while Cambodia had the lowest number (226 cases). The high incidence of HBV and the lack of success of national immunisation programmes and schemes are the most likely causes of the high prevalence of HCC in this country [72,73,74,75]. The extensive range of HCC case counts reflects the regional differences and heterogeneity in the incidence and consequences of HBV-related HCC in Southeast Asia [76]. The estimates in this study put the combined prevalence of HCC among HBV cases in this area at 45.8%. The high rate of hepatitis B infection in Southeast Asia and its accompanying comorbidities and consequences can be blamed for the high burden of HCC in HBV cases there. The results of this study agree with those of earlier studies [77,78,79,80].

Our study revealed a considerable concentration of publications in 2016 up to this point, which is an intriguing finding. The collective effort and study interest during that time are highlighted by this temporal trend, which may be a sign of significant developments and new information [81]. The results of this investigation corroborate previous reports [82]. The introduction of an effective HBV vaccine in the late 1980s could be the probable reason for the drop in HCC incidence at the start of the 20th century. Further, the advancement in HBV treatment options could also be attributed to the reduction of HBV and HCC incidence in these countries. However, there is evidence of HBV resistance to some of the current therapeutic options [82]. Advances in the diagnostic method of HBV tremendously improved at the start of the 20th century, with rapid and more efficient diagnosis geared towards HBV surveillance and management, thereby reducing the associated morbidity. The reduction in the overall prevalence of HCC over the years, particularly the transition from the 1990s to the 2000s as observed in this study, can be attributed to the aforementioned factors.

This systematic review and meta-analysis comprised papers that were cross-sectional, retrospective, and case-control studies. Cross-sectional studies had the highest representation of these designs, with 21 articles. The likely cause of this is unknown, but it can be attributed to the growing interest in the HBV cohort, the high index of HBV mutations, and the likelihood that HBV will advance to liver cancer and cirrhosis. The latter is consistent with other people’s reports [19,47,83,84,85]. Each study design makes a significant contribution to the overall prevalence while also providing unique viewpoints on the connection between HBV and HCC in the Southeast Asian context [86,87].

A forest plot of the pooled prevalence is shown in Figure 2, illustrating the significant burden of HCC in HBV cases in Southeast Asia. There was no publication bias among the recruited studies; the probable reason for this could be due to the quality of the recruited studies and the variability and dynamics of the recruited studies. The results of this study complement the reports of Zhu et al. (2016) and Wiangnon et al. (2012) [88,89]. Additionally, a funnel plot was created (Figure 3) to evaluate the possibility of publication bias in the pooled prevalence estimates of HCC and HBV in Southeast Asia. The plot shows a symmetrical distribution of studies around the pooled prevalence, indicating no publication bias, which increases confidence in the derived estimates.

This study’s subgroup meta-analysis showed that the pooled prevalence estimates varied across different nations, highlighting the complexity of HBV-related HCC in Southeastern countries [88]. Thailand distinguished itself among the nations examined with the most research, totalling 19. The recent rise in the morbidity of HBV-associated malignancies may be because of many reports from Thailand. Despite having only eight studies available for analysis, Singapore had the highest pooled prevalence of HCC in HBV cases, estimated at 62.5%. The high prevalence of HCC in Singapore may be related to the high incidence of HCC in HBV cases in Singapore; our report is consistent with the findings of others [45,90]. With a prevalence estimate of 40.9%, Cambodia showed the lowest prevalence. The prevalence estimate was, however, from only one study. Furthermore, Vietnam, Singapore, Thailand, the Philippines, Indonesia, and Malaysia were found to have high levels of heterogeneity. This shows a wide range in the prevalence estimates within these nations, pointing to the importance of several variables such as demographic traits, healthcare systems, and study methodology [91,92].

The highest pooled prevalence estimate of HCC among HBV cases within Southeast Asia was found in cross-sectional studies, which was estimated at 53.0%. This suggests that HCC is more common among HBV cases discovered by cross-sectional research. The likely cause of the increased prevalence could be related to the recent rise in interest in complications of HBV-associated liver illness [93,94,95]. Retrospective study designs showed the lowest pooled prevalence estimate of HCC in HBV cases within this group, estimated at 36.5%, despite having 14 included studies. The probable reason for the latter is unclear, but it could be attributed to the ongoing monitoring and surveillance requirement, which is not present in retrospective research [96].

Studies published between 1976 and 2022 (46 years) were included in the systematic review and meta-analysis. The latest decade (2016–present), which included 14 research studies, had the most publications of all the year groupings. Interestingly, the latest decade showed the lowest pooled prevalence estimate of HCC in HBV cases across Southeast Asia, estimated at 37.6%, despite the increased number of studies. The results imply that there may not be a linear relationship between the prevalence of HCC and HBV cases in Southeast Asia over time. Even though there have been more studies in recent years, the prevalence estimates were lower than in the past. This may be due to several factors, including shifts in population demographics, improvements in medical procedures, risk factors, or developing research methodology [76,97,98,99,100,101]. Lim et al. (2021) and Nguyen-Dinh et al. (2018) both reported cases of HBV in Singapore and Vietnam. The probable reason for the HBV incidence in these countries could be attributed to the high susceptibility index of people in these nations to HBV [69,70].

The meta-analysis encompasses a diverse range of studies involving both males and females. Remarkably, the prevalence of the condition among males is more than double that of females, with an estimated prevalence of 18.6% (95% CI: 14.3–23.9%). The probable reason for the low incidence of HCC in females is unclear, but it could be attributed to the high titer level of tumour-associated hormones and biomarkers dominant in males. This finding is in line with the report of Sizaret et al. (1975) [102], who reported that gender is an important associated factor in the distribution pattern of HCC. The meta-analysis also features three genotypes (i.e., A, B, and C) with a similar prevalence of 0.3%. The probable reason for the low prevalence of HCC in relation to HBV genotypes is unclear, but it could be attributed to the low genotypic reports within our study cohorts. The findings of this study establish a correlation between HCC acquisition and HBV genotypes. The latter is in agreement with the reports of others [13,103,104].

Cirrhosis has been established to be a significant factor in HCC development and progression [9]. In line with this, this study found a higher prevalence (6.7%) in individuals with cirrhosis compared to those without the condition (4.1%). The findings of this study are in line with the reports of others [9], [15,65,105,106]. Based on the stages of HCC, there was a higher prevalence of HCC in individuals with an early disease stage than in those with an intermediate or late stage. The findings of this study correspond with those of other reports [81,107], which independently establish that the stage of HCC is essential in HCC management and treatment.

### Strengths and Limitations of the Study

This study’s systematic review and meta-analysis have several strengths and a few drawbacks. One of the strengths is the comprehensive analysis of available data, which encompasses several Southeast Asian countries. To the best of our knowledge, this is the first systematic review and meta-analysis to report the prevalence of HCC in HBV patients, specifically within Southeast Asia, providing valuable insights into this region. A notable limitation, however, is the unavailability of data from certain Southeast Asian countries that met our inclusion criteria. For example, Cambodia had only one study representing a single cohort of HCC cases among the HBV population. Furthermore, there was an unavailability of data from countries like Myanmar, Brunei, East Timor, and Laos. This could introduce bias and impact the overall pooled prevalence estimate.

## 5. Conclusions

With a pooled prevalence estimate of 45.8%, this systematic review and meta-analysis show a significant prevalence of HCC in the HBV community in Southeast Asia. It is interesting to note, however, that Southeast Asia has shown a substantial decline in the combined prevalence of HCC and HBV over the past ten years. This trend raises the possibility that HCC will become less common among HBV carriers. These findings have important implications for government officials, countries, and organizations. Given the overall high frequency of HCC in HBV cases, there is a critical need for efficient preventative measures, early diagnostic techniques, and all-encompassing management plans. Policymakers can create focused interventions and devote resources to lessening the burden of morbidity associated with HCC in the HBV population in Southeast Asia and the Asian continent at large.

## Figures and Tables

**Table 1:** Characteristics of all the included studies.

Study ID	Year	Country	Total Number of HBV Cases	Positive HCC	Type of Study	Average Age (Range)	Gender		Positive HBV Genotypes			Cirrhosis		Stage of HCC			Treatment Status		AFP Detectable Level	
							Male	Female	A	B	C	Yes	No	Early	Intermediate	Late	Treated	Naïve	Yes	No
Pongpipat et al. [33]	1983	Thailand	17	13	Cross-sectional	7.4 (1–14)	NR	NR	NR	NR	NR	NR	NR	NR	NR	NR	8	5	8	5
Doury et al. [34]	1978	Vietnam	25	16	Cross-sectional	NR	NR	NR	NR	NR	NR	NR	NR	NR	NR	NR	NR	NR	NR	NR
Chan et al. [35]	1980	Singapore	18	17	Cross-sectional	NR	NR	NR	NR	NR	NR	1	16	NR	NR	NR	NR	NR	11	6
Nun-Anan et al. [36]	2015	Thailand	50	19	Cross-sectional	56.4 (18–78)	13	6	NR	NR	NR	1	18	11	6	2	NR	NR	NR	NR
Kamalapirat et al. [37]	2021	Thailand	2208	20	Cross-sectional	41.36 (18–82)	11	9	NR	NR	NR	3	17	7	9	4	NR	NR	NR	NR
Wungu et al. [38]	2018	Indonesia	32	21	Cross-sectional	NR	NR	NR	NR	NR	NR	NR	NR	NR	NR	NR	NR	NR	NR	NR
Tangkijvanich et al. [39]	2001	Thailand	72	22	Cross-sectional	NR	NR	NR	NR	NR	NR	11	11	6	13	3	7	15	NR	NR
Nørredam et al. [40]	1986	Thailand	37	24	Cross-sectional	50.3 (18–81)	11	13	NR	NR	NR	16	8	NR	NR	NR	NR	NR	NR	NR
Tongsiri et al. [41]	2017	Thailand	396	26	Retrospective	56.3 (18–76)	18	8	NR	NR	NR	9	17	14	5	7	NR	NR	21	5
Sakamoto et al. [42]	2006	Philippines	100	31	Cross-sectional	53.7 (18–73)	26	5	11	11	9	25	6	12	9	10	27	4	NR	NR
Tan et al. [43]	1977	Singapore	50	37	Cross-sectional	NR	NR	NR	NR	NR	NR	27	10	NR	NR	NR	NR	NR	26	11
Wanich et al. [44]	2016	Thailand	164	38	Cross-sectional	59.6 (18–87)	31	7	NR	NR	NR	28	10	20	6	12	29	9	23	15
Khin et al. [45]	1996	Singapore	55	41	Case control	NR	NR	NR	NR	NR	NR	NR	NR	NR	NR	NR	NR	NR	NR	NR
Jack et al. [46]	2019	Vietnam	362	49	Retrospective	NR	NR	NR	NR	NR	NR	38	11	15	20	14	37	12	41	8
Huong et al. [47]	2022	Vietnam	247	49	Retrospective	57.9 (18–81)	34	15	1	42	5	32	17	NR	NR	NR	NR	NR	NR	NR
Pramoolsinsap et al. [8]	1992	Thailand	168	50	Case control	50.8 (19–79)	31	19	NR	NR	NR	NR	NR	NR	NR	NR	NR	NR	NR	NR
Irie et al. [48]	1985	Philippines	150	56	Retrospective	NR	NR	NR	NR	NR	NR	NR	NR	NR	NR	NR	NR	NR	NR	NR
Chao et al. [49]	1994	Singapore	423	58	Retrospective	NR	NR	NR	NR	NR	NR	NR	NR	NR	NR	NR	NR	NR	NR	NR
Lim et al. [50]	1986	Singapore	77	61	Cross-sectional	NR	55	6	NR	NR	NR	NR	NR	NR	NR	NR	NR	NR	61	0
Welsh et al. [51]	1976	Vietnam	306	61	Cross-sectional	NR	NR	NR	NR	NR	NR	NR	NR	NR	NR	NR	NR	NR	NR	NR
Tangkijvanich et al. [52]	1999	Thailand	101	66	Cross-sectional	54.4 (18–79)	48	18	NR	NR	NR	19	47	NR	NR	NR	NR	NR	NR	NR
Sulaiman and ﻿Sulaiman [53]	1989	Indonesia	226	82	Retrospective	51.4 (21–78)	68	14	NR	NR	NR	29	53	NR	NR	NR	20	62	NR	NR
Pramoolsinsap et al. [54]	1994	Thailand	98	83	Cross-sectional	49.2 (18–81)	51	32	NR	NR	NR	21	62	NR	NR	NR	NR	NR	NR	NR
Tangkijvanich et al. [55]	2003	Thailand	188	105	Retrospective	42.9 (18–73)	NR	NR	NR	NR	NR	NR	NR	NR	NR	NR	NR	NR	NR	NR
Norsa’adah et al. [56]	2013	Malaysia	210	121	Cross-sectional	55 (16–82)	98	23	NR	NR	NR	42	79	40	58	23	102	19	121	0
Tangkijvanich et al. [57]	1999	Thailand	200	130	Cross-sectional	NR	NR	NR	NR	NR	NR	NR	NR	NR	NR	NR	NR	NR	NR	NR
Makkoch et al. [58]	2016	Thailand	369	141	Cross-sectional	51.8 (18–82)	98	43	NR	NR	NR	NR	NR	NR	NR	NR	NR	NR	NR	NR
Omar et al. [59]	2015	Malaysia	348	143	Cross-sectional	56.2 (17–85)	99	44	NR	NR	NR	NR	NR	52	47	44	NR	NR	NR	NR
Sooklim et al. [5]	2003	Thailand	180	147	Retrospective	NR	NR	NR	NR	NR	NR	NR	NR	NR	NR	NR	NR	NR	NR	NR
Somboonet et al. [6]	2014	Thailand	308	153	Retrospective	57.4 (18–75)	99	54	NR	NR	NR	146	7	50	49	54	97	56	NR	NR
Hoan et al. [60]	2019	Vietnam	443	171	Case control	51 (18–90)	129	42	NR	NR	NR	117	54	NR	NR	NR	NR	NR	NR	NR
Chanthra et al. [61]	2016	Thailand	582	192	Case control	57.6 (18–83)	164	28	NR	NR	NR	NR	NR	NR	NR	NR	NR	NR	NR	NR
Sopipong et al. [62]	2013	Thailand	398	202	Case control	59.8 (18–81)	158	44	NR	NR	NR	NR	NR	NR	NR	NR	NR	NR	NR	NR
Poh et al. [63]	2015	Singapore	673	209	Case control	56.3 (17–82)	134	75	NR	NR	NR	154	55	NR	NR	NR	NR	NR	NR	NR
Chassagne et al. [64]	2016	Cambodia	553	226	Retrospective	58.1 (28–91)	184	42	NR	NR	NR	196	30	NR	NR	NR	NR	NR	201	25
Lingao [65]	1989	Phillipines	340	254	Cross-sectional	46.1 (18–78)	198	56	NR	NR	NR	99	155	NR	NR	NR	NR	NR	249	5
Pawarode et al. [66]	2000	Thailand	368	258	Retrospective	52.1 (2–85)	190	68	NR	NR	NR	211	47	NR	NR	NR	248	10	NR	NR
Sriprapun et al. [67]	2016	Thailand	325	266	Cross-sectional	50.28 (18–91)	177	89	NR	NR	NR	80	186	94	120	52	NR	NR	NR	NR
Liew et al. [68]	2019	Singapore	1079	916	Retrospective	59.4 (2–93)	768	148	NR	NR	NR	NR	NR	197	556	163	NR	NR	NR	NR
Lim et al. [69]	2021	Singapore	3013	1404	Retrospective	63.8 (18–89)	1028	376	NR	NR	NR	NR	NR	NR	NR	NR	787	617	NR	NR
Nguyen-Dinh et al. [70]	2018	Vietnam	24,091	1501	Retrospective	NR	NR	NR	NR	NR	NR	NR	NR	NR	NR	NR	NR	NR	NR	NR

**Table 2:** Subgroup analysis of HCC in HBV cases within Southeast Asia in relation to assessed parameters.

Subgroup Meta-Analysis	Number of Studies	Prevalence of HCC (%)	95% CI	*I*^2^ (%)	Q	Heterogeneity Test	
						D.F	*p*
**Country**							
Vietnam	6	22.9	9.7–44.9	99.27	682.60	5	<0.001
Singapore	8	62.5	42.4–79.1	99.05	739.37	7	<0.001
Thailand	19	45.7	32.9–59.1	98.3	1058.90	18	<0.001
Philippines	3	48.2	21.0–76.5	97.75	88.72	2	<0.001
Indonesia	2	49.8	23.3–76.4	89.23	9.28	1	0.002
Malaysia	2	49.2	33.5–65.1	92.96	14.20	1	<0.001
Cambodia	1	40.9	36.8–45.0	-	-	-	-
**Study design**							
Cross-sectional	21	53.0	39.1–66.5	97.86	935.81	20	<0.001
Retrospective	14	36.5	18.7–59.0	99.79	6117.67	13	<0.001
Case-control	6	41.2	32.9–50.0	99.51	76.68	5	<0.001
**Year of publication**							
1975–1985	7	58.0	37.1–76.4	97.66	256.53	6	<0.001
1986–1995	6	47.1	30.4–64.5	96.87	159.99	5	<0.001
1996–2005	7	48.9	39.7–58.3	92.1	75.95	6	<0.001
2006–2015	7	44.1	22.4–68.2	98.08	312.99	6	<0.001
2016–2023	14	37.6	19.2–60.5	99.78	6039.49	6	<0.001
**Gender**							
Male	26	18.6	14.3–23.9	97.33	562.31	25	<0.001
Female	26	6.9	5.4–8.8	91.06	496.64	25	<0.001
**HBV genotypes**							
A	2	0.3	0.1–0.6	72.04	158.38	1	<0.001
B	2	0.3	0.1–0.7	86.74	212.96	1	<0.001
C	2	0.3	0.1–0.6	68.12	135.27	1	<0.001
**Cirrhosis**							
Yes	22	6.7	4.7–9.5	95.17	763.82	21	<0.001
No	22	4.1	2.5–6.8	96.96	647.47	21	<0.001
**Stages of HCC**							
Early	12	4.1	2.5–6.8	96.96	339.72	11	<0.001
Intermediate	12	1.1	0.6–2.0	96.41	281.57	11	<0.001
Late	12	1.3	0.8–2.0	90.18	407.15	11	<0.001
**AFP detectabble level**							
Yes	10	0.7	0.5–1.0	98.03	312.17	9	<0.001
No	10	0.6	0.3–1.2	88.07	269.04	9	<0.001

## Data Availability

The data availability statement is not applicable for the current review.

## Supplementary Materials

The following supporting information can be downloaded at: https://www.mdpi.com/article/10.3390/pathogens12101220/s1. File S1: Search strategy of HCC in HBV cohort within southeast Asia. File S2: JBI checklist for prevalence data. File S3: PRISMA guidelines. File S4: Subgroup by year of study HCC in HBV. Table S1: Quality of included studies by the JBJ critical appraisal checklist for studies reporting prevalence data.
