# Supplementary material for: Prevalence of Hepatocellular Carcinoma in Hepatitis B Population within Southeast Asia: A Systematic Review and Meta-Analysis of 39,050 Participants
Source: Pathogens. 2023 Oct 6;12(10):1220. doi: 10.3390/pathogens12101220 (PMC10609743; doi:10.3390/pathogens12101220)
Supplement: Supplementary file 1 [file pathogens-12-01220-s001.zip › File S1 SEARCH STRATEGY OF HCC IN HBV COHORT WITHIN SOUTHEAST ASIA.pdf]

SEARCH STRATEGY

SCOPUS

(((TITLE-ABS-KEY(hepatocellular carcinoma)) OR (TITLE-ABS-KEY(HCC))) OR (TITLE-ABS-KEY(hepatocyte carcinoma)) OR (TITLE-ABS-KEY(liver cancer))) AND ((TITLE-ABS-KEY(hepatitis C)) OR (TITLE-ABS-KEY(hepatitis C virus))) AND (((TITLE-ABS-KEY(Brunei)) OR (TITLE-ABS-KEY(Cambodia)) OR (TITLE-ABS-KEY(Indonesia)) OR (TITLE-ABS-KEY(Laos))) OR ((TITLE-ABS-KEY(Brunei)) OR (TITLE-ABS-KEY(Cambodia)) OR (TITLE-ABS-KEY(Indonesia))) OR ((TITLE-ABS-KEY(Laos)) OR (TITLE-ABS-KEY(Malaysia)) OR (TITLE-ABS-KEY(Myanmar)) OR (TITLE-ABS-KEY(Burma))) OR ((TITLE-ABS-KEY(Philippines)) OR (TITLE-ABS-KEY(Singapore)) OR (TITLE-ABS-KEY(Thailand)) OR (TITLE-ABS-KEY(Timor-Leste)) OR (TITLE-ABS-KEY(East Timor)) OR (TITLE-ABS-KEY(Vietnam)) OR (TITLE-ABS-KEY("Timor leste")))).

HCC PUBMED

("brunei"[MeSH Terms] OR "brunei"[All Fields] OR ("cambodia"[MeSH Terms] OR "cambodia"[All Fields] OR "cambodia s"[All Fields]) OR ("indonesia"[MeSH Terms] OR "indonesia"[All Fields] OR "indonesia s"[All Fields] OR "indonesias"[All Fields]) OR ("brunei"[MeSH Terms] OR "brunei"[All Fields]) OR ("laos"[MeSH Terms] OR "laos"[All Fields]) OR ("malaysia"[MeSH Terms] OR "malaysia"[All Fields] OR "malaysia s"[All Fields]) OR ("myanmar"[MeSH Terms] OR "myanmar"[All Fields] OR "myanmar s"[All Fields] OR "myanmars"[All Fields]) OR ("burma s"[All Fields] OR "myanmar"[MeSH Terms] OR "myanmar"[All Fields] OR "burma"[All Fields]) OR ("philippine"[All Fields] OR "philippines"[MeSH Terms] OR "philippines"[All Fields]) OR ("singapore"[MeSH Terms] OR "singapore"[All Fields] OR "singapore s"[All Fields]) OR ("thailand"[MeSH Terms] OR "thailand"[All Fields] OR "thailand s"[All Fields]) OR ("Timor leste"[MeSH Terms] OR "Timor leste"[All Fields] OR ("timor"[All Fields] AND "leste"[All Fields]) OR "Timor leste"[All Fields]) OR ("Timor leste"[MeSH Terms] OR "Timor leste"[All Fields] OR ("east"[All Fields] AND "timor"[All Fields]) OR "east timor"[All Fields]) OR ("vietnam"[MeSH Terms] OR "vietnam"[All

Fields] OR "vietnam s"[All Fields]) OR "Timor leste"[All Fields]) AND ("hepatocellular carcinoma"[All Fields] OR ("liver neoplasms"[MeSH Terms] OR ("liver"[All Fields] AND "neoplasms"[All Fields]) OR "liver neoplasms"[All Fields] OR ("liver"[All Fields] AND "cancer"[All Fields]) OR "liver cancer"[All Fields]) OR "HCC"[All Fields]).

GOOGLE SCHOLAR

allintitle: Hepatocellular carcinoma AND hepatitis B AND Malaysia OR Singapore OR Thailand OR Cambodia OR Indonesia OR Myanmar OR Brunei OR East Timor OR Laos OR Vietnam OR Philippines "hepatocellular carcinoma" "Hepatitis B"

SCIENCE DIRECT

(Hepatocellular carcinoma OR liver cancer OR HCC) AND (Hepatitis B OR HBV) AND (Malaysia OR Singapore OR Thailand OR Cambodia OR Indonesia OR Myanmar OR Brunei OR East Timor OR Laos OR Vietnam OR Philippines)
