# Supplementary material for: Prevalence of Hepatocellular Carcinoma in Hepatitis B Population within Southeast Asia: A Systematic Review and Meta-Analysis of 39,050 Participants
Source: Pathogens. 2023 Oct 6;12(10):1220. doi: 10.3390/pathogens12101220 (PMC10609743; doi:10.3390/pathogens12101220)

MALE

**Studies**	Estimate (95% C.I.)	Ev/Trt
Pongpipat et al 1983	0.028 (0.002, 0.322)	0/17
Doury et al 1978	0.019 (0.001, 0.244)	0/25
Chan et al 1980	0.026 (0.002, 0.310)	0/18
Nun-Anan et al 2015	0.260 (0.157, 0.398)	13/50
Kamalapirat et al 2021	0.005 (0.003, 0.009)	11/2208
Wungu et al 2018	0.015 (0.001, 0.201)	0/32
Tang.kijvanich et al 2001	0.007 (0.000, 0.100)	0/72
Nørredam et al 1986	0.297 (0.173, 0.461)	11/37
Tongsiri et al 2017	0.045 (0.029, 0.071)	18/396
Sakamoto et al 2006	0.260 (0.184, 0.355)	26/100
Tan et al 1977	0.010 (0.001, 0.138)	0/50
Wanich et al 2016	0.189 (0.136, 0.256)	31/164
Khin et al 1996	0.009 (0.001, 0.127)	0/55
Jack et al 2019	0.001 (0.000, 0.022)	0/362
Huong et 2022	0.138 (0.100, 0.186)	34/247
Pramoolsinsap et al 1992	0.185 (0.133, 0.250)	31/168
Irie et al 1985	0.003 (0.000, 0.051)	0/150
Chao et al 1994	0.001 (0.000, 0.019)	0/423
Lim et al 1986	0.922 (0.837, 0.965)	71/77
Welsh et al 1976	0.002 (0.000, 0.025)	0/306
T.angkijvanich et al 1999	0.475 (0.380, 0.572)	48/101
Sulaiman and Sulaiman 1989	0.301 (0.245, 0.364)	68/226
Pramoolsins.ap et al 1994	0.520 (0.422, 0.617)	51/98
Tangkijvanich et al 2003	0.003 (0.000, 0.041)	0/188
Norsa'adah et al 2013	0.467 (0.400, 0.534)	98/210
Tangkijvanic..h et al 1999	0.002 (0.000, 0.038)	0/200
Makkoch et al 2016	0.266 (0.223, 0.313)	98/369
Omar et al 2015	0.284 (0.240, 0.334)	99/348
Sooklim et al 2003	0.003 (0.000, 0.043)	0/180
Somboon et al 2014	0.321 (0.272, 0.376)	99/308
Hoan et al 2019	0.291 (0.251, 0.335)	129/443
Chanthra et al 2016	0.282 (0.247, 0.320)	164/582
Sopipong et al 2013	0.397 (0.350, 0.446)	158/398
Poh et al 2015	0.199 (0.171, 0.231)	134/673
Chassagne et al 2016	0.333 (0.295, 0.373)	184/553
Lingao 1989	0.582 (0.529, 0.634)	198/340
Pawarode et al 2000	0.516 (0.465, 0.567)	190/368
Sriprapun et al 2016	0.545 (0.490, 0.598)	177/325
Liew et al 2019	0.712 (0.684, 0.738)	768/1079
Lim et al 2021	0.341 (0.324, 0.358)	1028/3013
Nguyen-Dinh et al 2018	0.000 (0.000, 0.000)	0/24091
**Overall (I^2=97.33 % , P< 0.001)**	**0.186 (0.143, 0.239)**	**3937/39050**

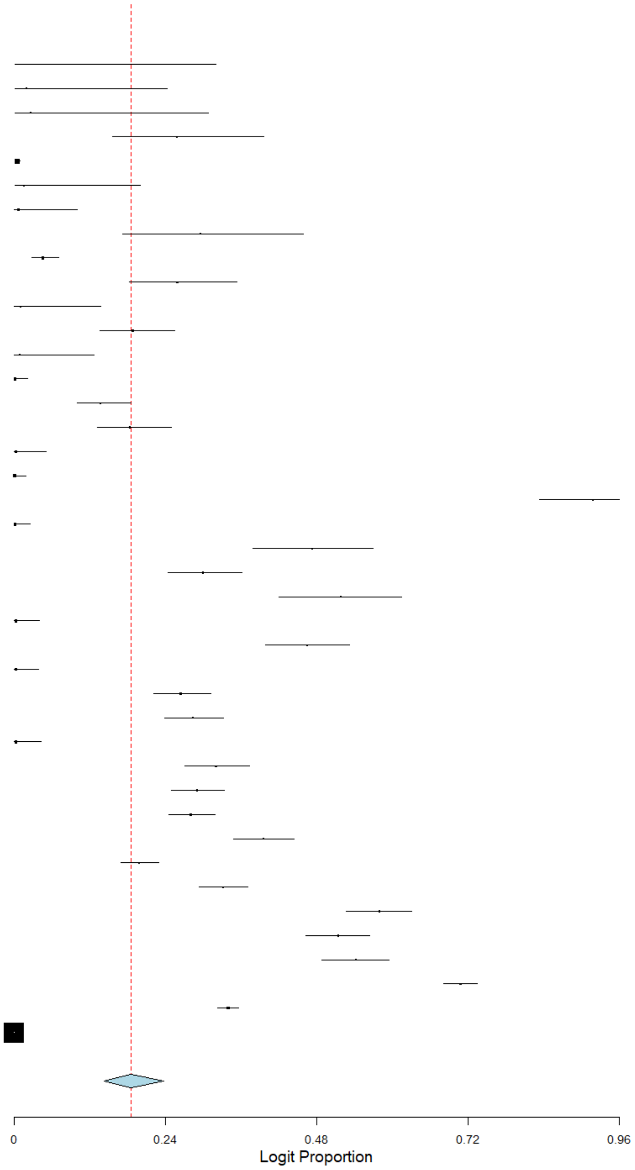

FEMALE

Studies	Estimate (95% C.I.)	Ev/Trt
Pongpipat et al 1983	0.028 (0.002, 0.322)	0/17
Doury et al 1978	0.019 (0.001, 0.244)	0/25
Chan et al 1980	0.026 (0.002, 0.310)	0/18
Nun-Anan et al 2015	0.120 (0.055, 0.242)	6/50
Kamalapirat et al 2021	0.004 (0.002, 0.008)	9/2208
Wungu et al 2018	0.015 (0.001, 0.201)	0/32
Tang.kijvanich et al 2001	0.007 (0.000, 0.100)	0/72
Nørredam et al 1986	0.486 (0.332, 0.644)	18/37
Tongsiri et al 2017	0.020 (0.010, 0.040)	8/396
Sakamoto et al 2006	0.050 (0.021, 0.115)	5/100
Tan et al 1977	0.010 (0.001, 0.138)	0/50
Wanich et al 2016	0.043 (0.020, 0.087)	7/164
Khin et al 1996	0.009 (0.001, 0.127)	0/55
Jack et al 2019	0.001 (0.000, 0.022)	0/362
Huong et 2022	0.061 (0.037, 0.098)	15/247
Pramoolsinsap et al 1992	0.113 (0.073, 0.171)	19/168
Irie et al 1985	0.003 (0.000, 0.051)	0/150
Chao et al 1994	0.001 (0.000, 0.019)	0/423
Lim et al 1986	0.078 (0.035, 0.163)	6/77
Welsh et al 1976	0.002 (0.000, 0.025)	0/306
T.angkijvanich et al 1999	0.178 (0.115, 0.265)	18/101
Sulaiman and Sulaiman 1989	0.062 (0.037, 0.102)	14/226
Pramoolsins.ap et al 1994	0.327 (0.241, 0.425)	32/98
Tangkijvanich et al 2003	0.003 (0.000, 0.041)	0/188
Norsa'adah et al 2013	0.110 (0.074, 0.159)	23/210
Tangkijvanic..h et al 1999	0.002 (0.000, 0.038)	0/200
Makkoch et al 2016	0.117 (0.088, 0.153)	43/369
Omar et al 2015	0.126 (0.095, 0.166)	44/348
Sooklim et al 2003	0.003 (0.000, 0.043)	0/180
Somboon et al 2014	0.175 (0.137, 0.222)	54/308
Hoan et al 2019	0.095 (0.071, 0.126)	42/443
Chanthra et al 2016	0.048 (0.033, 0.069)	28/582
Sopipong et al 2013	0.111 (0.083, 0.145)	44/398
Poh et al 2015	0.111 (0.090, 0.138)	75/673
Chassagne et al 2016	0.087 (0.066, 0.113)	48/553
Lingao 1989	0.165 (0.129, 0.208)	56/340
Pawarode et al 2000	0.185 (0.148, 0.228)	68/368
Sriprapun et al 2016	0.028 (0.014, 0.052)	9/325
Liew et al 2019	0.137 (0.118, 0.159)	148/1079
Lim et al 2021	0.125 (0.113, 0.137)	376/3013
Nguyen-Dinh et al 2018	0.000 (0.000, 0.000)	0/24091
**Overall (I^2=91.06 % , P< 0.001)**	**0.069 (0.054, 0.088)**	**1215/39050**

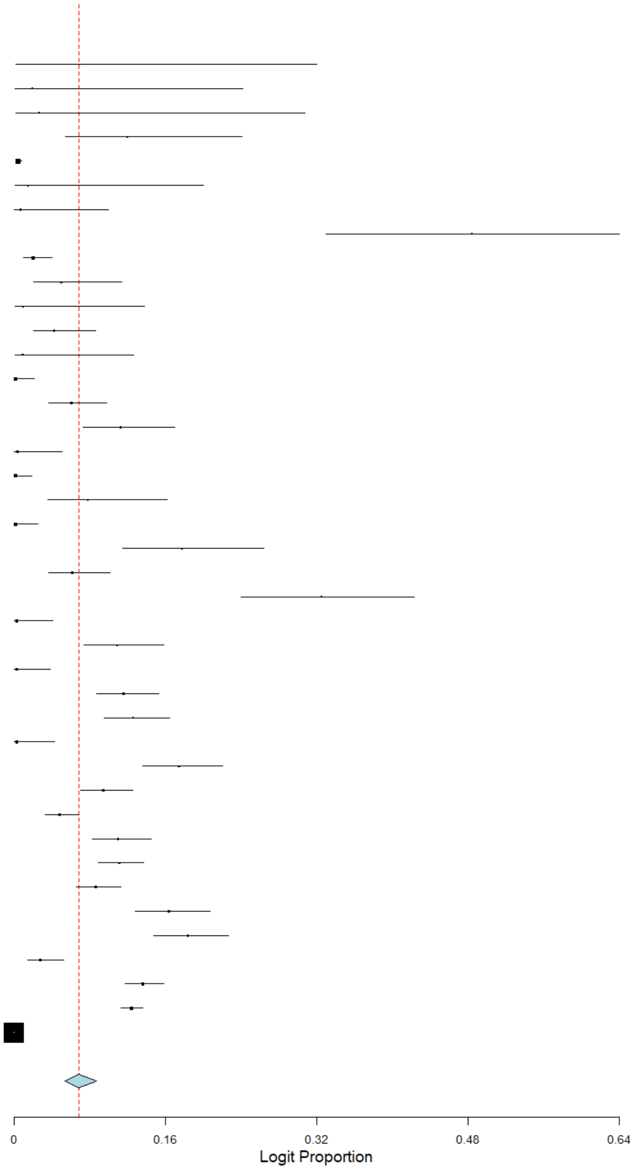

GENOTYPE A

Studies	Estimate (95% C.I.)	Ev/Trt
Pongpipat et al 1983	0.028 (0.002, 0.322)	0/17
Doury et al 1978	0.019 (0.001, 0.244)	0/25
Chan et al 1980	0.026 (0.002, 0.310)	0/18
Nun-Anan et al 2015	0.010 (0.001, 0.138)	0/50
Kamalapirat et al 2021	0.000 (0.000, 0.004)	0/2208
Wungu et al 2018	0.015 (0.001, 0.201)	0/32
Tang.kijvanich et al 2001	0.007 (0.000, 0.100)	0/72
Nørredam et al 1986	0.013 (0.001, 0.178)	0/37
Tongsiri et al 2017	0.001 (0.000, 0.020)	0/396
Sakamoto et al 2006	0.110 (0.062, 0.188)	11/100
Tan et al 1977	0.010 (0.001, 0.138)	0/50
Wanich et al 2016	0.003 (0.000, 0.047)	0/164
Khin et al 1996	0.009 (0.001, 0.127)	0/55
Jack et al 2019	0.001 (0.000, 0.022)	0/362
Huong et 2022	0.004 (0.001, 0.028)	1/247
Pramoolsinsap et al 1992	0.003 (0.000, 0.045)	0/168
Irie et al 1985	0.003 (0.000, 0.051)	0/150
Chao et al 1994	0.001 (0.000, 0.019)	0/423
Lim et al 1986	0.006 (0.000, 0.094)	0/77
Welsh et al 1976	0.002 (0.000, 0.025)	0/306
T.angkijvanich et al 1999	0.005 (0.000, 0.073)	0/101
Sulaiman and Sulaiman 1989	0.002 (0.000, 0.034)	0/226
Pramoolsins.ap et al 1994	0.005 (0.000, 0.076)	0/98
Tangkijvanich et al 2003	0.003 (0.000, 0.041)	0/188
Norsa'adah et al 2013	0.002 (0.000, 0.037)	0/210
Tangkijvanic..h et al 1999	0.002 (0.000, 0.038)	0/200
Makkoch et al 2016	0.001 (0.000, 0.021)	0/369
Omar et al 2015	0.001 (0.000, 0.022)	0/348
Sooklim et al 2003	0.003 (0.000, 0.043)	0/180
Somboon et al 2014	0.002 (0.000, 0.025)	0/308
Hoan et al 2019	0.001 (0.000, 0.018)	0/443
Chanthra et al 2016	0.001 (0.000, 0.014)	0/582
Sopipong et al 2013	0.001 (0.000, 0.020)	0/398
Poh et al 2015	0.001 (0.000, 0.012)	0/673
Chassagne et al 2016	0.001 (0.000, 0.014)	0/553
Lingao 1989	0.001 (0.000, 0.023)	0/340
Pawarode et al 2000	0.001 (0.000, 0.021)	0/368
Sriprapun et al 2016	0.002 (0.000, 0.024)	0/325
Liew et al 2019	0.000 (0.000, 0.007)	0/1079
Lim et al 2021	0.000 (0.000, 0.003)	0/3013
Nguyen-Dinh et al 2018	0.000 (0.000, 0.000)	0/24091
**Overall (I^2=72.04 % , P< 0.001)**	**0.003 (0.001, 0.006)**	**12/39050**

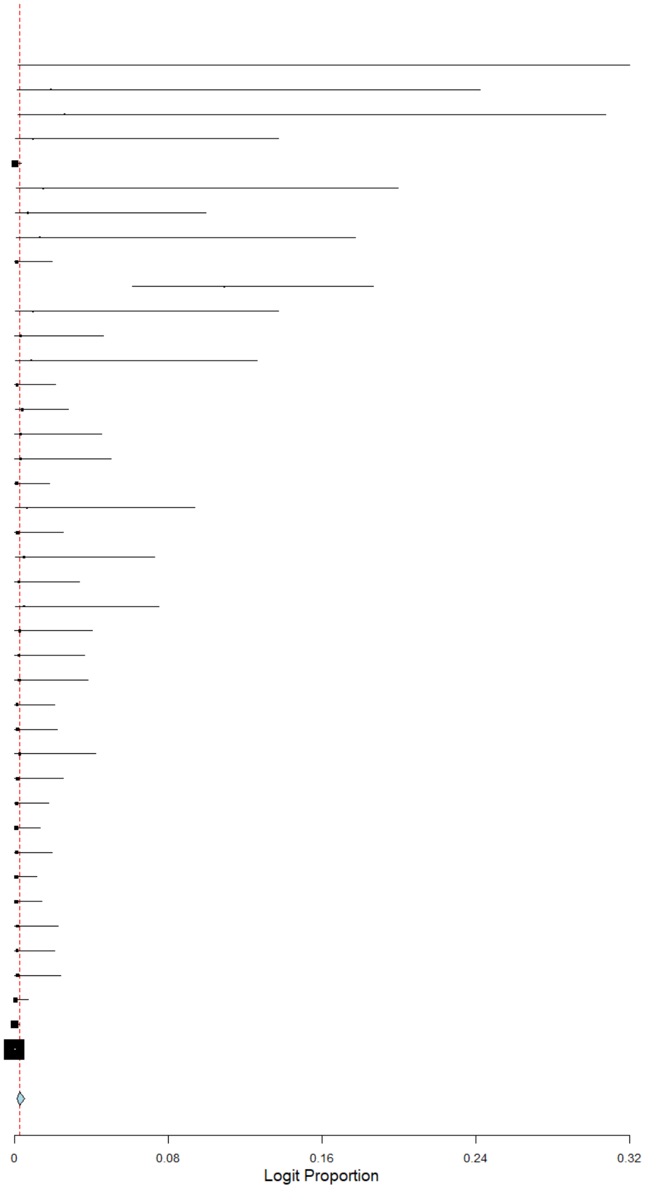

GENOTYPE B

Studies	Estimate (95% C.I.)	Ev/Trt
Pongpipat et al 1983	0.028 (0.002, 0.322)	0/17
Doury et al 1978	0.019 (0.001, 0.244)	0/25
Chan et al 1980	0.026 (0.002, 0.310)	0/18
Nun-Anan et al 2015	0.010 (0.001, 0.138)	0/50
Kamalapirat et al 2021	0.000 (0.000, 0.004)	0/2208
Wungu et al 2018	0.015 (0.001, 0.201)	0/32
Tang.kijvanich et al 2001	0.007 (0.000, 0.100)	0/72
Nørredam et al 1986	0.013 (0.001, 0.178)	0/37
Tongsiri et al 2017	0.001 (0.000, 0.020)	0/396
Sakamoto et al 2006	0.110 (0.062, 0.188)	11/100
Tan et al 1977	0.010 (0.001, 0.138)	0/50
Wanich et al 2016	0.003 (0.000, 0.047)	0/164
Khin et al 1996	0.009 (0.001, 0.127)	0/55
Jack et al 2019	0.001 (0.000, 0.022)	0/362
Huong et 2022	0.170 (0.128, 0.222)	42/247
Pramoolsinsap et al 1992	0.003 (0.000, 0.045)	0/168
Irie et al 1985	0.003 (0.000, 0.051)	0/150
Chao et al 1994	0.001 (0.000, 0.019)	0/423
Lim et al 1986	0.006 (0.000, 0.094)	0/77
Welsh et al 1976	0.002 (0.000, 0.025)	0/306
T.angkijvanich et al 1999	0.005 (0.000, 0.073)	0/101
Sulaiman and Sulaiman 1989	0.002 (0.000, 0.034)	0/226
Pramoolsins.ap et al 1994	0.005 (0.000, 0.076)	0/98
Tangkijvanich et al 2003	0.003 (0.000, 0.041)	0/188
Norsa'adah et al 2013	0.002 (0.000, 0.037)	0/210
Tangkijvanic..h et al 1999	0.002 (0.000, 0.038)	0/200
Makkoch et al 2016	0.001 (0.000, 0.021)	0/369
Omar et al 2015	0.001 (0.000, 0.022)	0/348
Sooklim et al 2003	0.003 (0.000, 0.043)	0/180
Somboon et al 2014	0.002 (0.000, 0.025)	0/308
Hoan et al 2019	0.001 (0.000, 0.018)	0/443
Chanthra et al 2016	0.001 (0.000, 0.014)	0/582
Sopipong et al 2013	0.001 (0.000, 0.020)	0/398
Poh et al 2015	0.001 (0.000, 0.012)	0/673
Chassagne et al 2016	0.001 (0.000, 0.014)	0/553
Lingao 1989	0.001 (0.000, 0.023)	0/340
Pawarode et al 2000	0.001 (0.000, 0.021)	0/368
Sriprapun et al 2016	0.002 (0.000, 0.024)	0/325
Liew et al 2019	0.000 (0.000, 0.007)	0/1079
Lim et al 2021	0.000 (0.000, 0.003)	0/3013
Nguyen-Dinh et al 2018	0.000 (0.000, 0.000)	0/24091
**Overall (I^2=86.74 % , P< 0.001)**	**0.003 (0.001, 0.007)**	**53/39050**

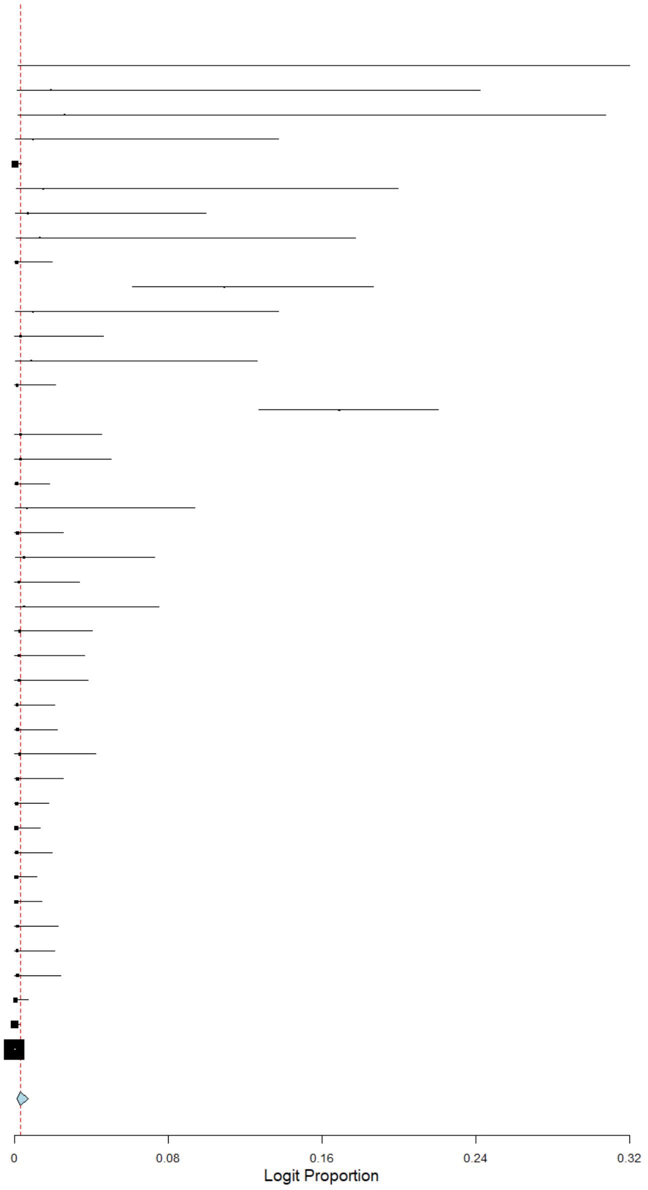

GENOTYPE C

Studies	Estimate (95% C.I.)	Ev/Trt
Pongpipat et al 1983	0.028 (0.002, 0.322)	0/17
Doury et al 1978	0.019 (0.001, 0.244)	0/25
Chan et al 1980	0.026 (0.002, 0.310)	0/18
Nun-Anan et al 2015	0.010 (0.001, 0.138)	0/50
Kamalapirat et al 2021	0.000 (0.000, 0.004)	0/2208
Wungu et al 2018	0.015 (0.001, 0.201)	0/32
Tang.kijvanich et al 2001	0.007 (0.000, 0.100)	0/72
Nørredam et al 1986	0.013 (0.001, 0.178)	0/37
Tongsiri et al 2017	0.001 (0.000, 0.020)	0/396
Sakamoto et al 2006	0.090 (0.047, 0.164)	9/100
Tan et al 1977	0.010 (0.001, 0.138)	0/50
Wanich et al 2016	0.003 (0.000, 0.047)	0/164
Khin et al 1996	0.009 (0.001, 0.127)	0/55
Jack et al 2019	0.001 (0.000, 0.022)	0/362
Huong et 2022	0.020 (0.008, 0.048)	5/247
Pramoolsinsap et al 1992	0.003 (0.000, 0.045)	0/168
Irie et al 1985	0.003 (0.000, 0.051)	0/150
Chao et al 1994	0.001 (0.000, 0.019)	0/423
Lim et al 1986	0.006 (0.000, 0.094)	0/77
Welsh et al 1976	0.002 (0.000, 0.025)	0/306
T.angkijvanich et al 1999	0.005 (0.000, 0.073)	0/101
Sulaiman and Sulaiman 1989	0.002 (0.000, 0.034)	0/226
Pramoolsins.ap et al 1994	0.005 (0.000, 0.076)	0/98
Tangkijvanich et al 2003	0.003 (0.000, 0.041)	0/188
Norsa'adah et al 2013	0.002 (0.000, 0.037)	0/210
Tangkijvanic..h et al 1999	0.002 (0.000, 0.038)	0/200
Makkoch et al 2016	0.001 (0.000, 0.021)	0/369
Omar et al 2015	0.001 (0.000, 0.022)	0/348
Sooklim et al 2003	0.003 (0.000, 0.043)	0/180
Somboon et al 2014	0.002 (0.000, 0.025)	0/308
Hoan et al 2019	0.001 (0.000, 0.018)	0/443
Chanthra et al 2016	0.001 (0.000, 0.014)	0/582
Sopipong et al 2013	0.001 (0.000, 0.020)	0/398
Poh et al 2015	0.001 (0.000, 0.012)	0/673
Chassagne et al 2016	0.001 (0.000, 0.014)	0/553
Lingao 1989	0.001 (0.000, 0.023)	0/340
Pawarode et al 2000	0.001 (0.000, 0.021)	0/368
Sriprapun et al 2016	0.002 (0.000, 0.024)	0/325
Liew et al 2019	0.000 (0.000, 0.007)	0/1079
Lim et al 2021	0.000 (0.000, 0.003)	0/3013
Nguyen-Dinh et al 2018	0.000 (0.000, 0.000)	0/24091
**Overall (I^2=68.12 % , P< 0.001)**	**0.003 (0.001, 0.006)**	**14/39050**

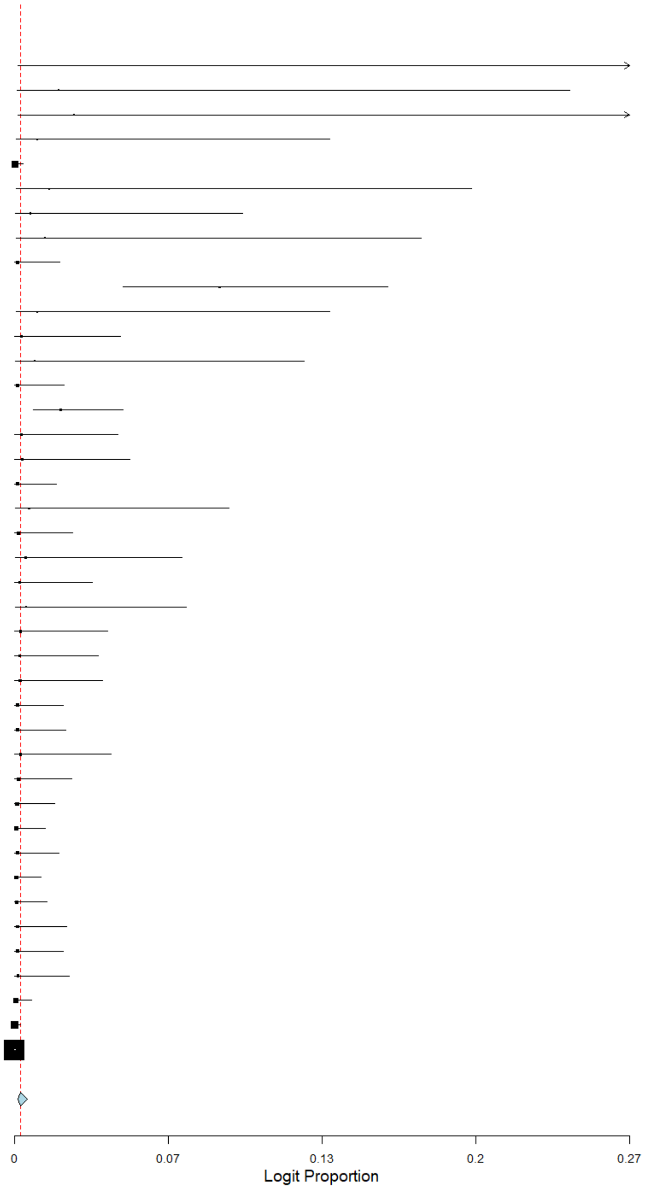

EARLY

Studies	Estimate (95% C.I.)	Ev/Trt
Pongpipat et al 1983	0.028 (0.002, 0.322)	0/17
Doury et al 1978	0.019 (0.001, 0.244)	0/25
Chan et al 1980	0.889 (0.648, 0.972)	16/18
Nun-Anan et al 2015	0.360 (0.240, 0.501)	18/50
Kamalapirat et al 2021	0.008 (0.005, 0.012)	17/2208
Wungu et al 2018	0.015 (0.001, 0.201)	0/32
Tang.kijvanich et al 2001	0.153 (0.087, 0.255)	11/72
Nørredam et al 1986	0.216 (0.112, 0.376)	8/37
Tongsiri et al 2017	0.043 (0.027, 0.068)	17/396
Sakamoto et al 2006	0.060 (0.027, 0.127)	6/100
Tan et al 1977	0.200 (0.111, 0.333)	10/50
Wanich et al 2016	0.061 (0.033, 0.110)	10/164
Khin et al 1996	0.009 (0.001, 0.127)	0/55
Jack et al 2019	0.030 (0.017, 0.054)	11/362
Huong et 2022	0.069 (0.043, 0.108)	17/247
Pramoolsinsap et al 1992	0.003 (0.000, 0.045)	0/168
Irie et al 1985	0.003 (0.000, 0.051)	0/150
Chao et al 1994	0.001 (0.000, 0.019)	0/423
Lim et al 1986	0.006 (0.000, 0.094)	0/77
Welsh et al 1976	0.002 (0.000, 0.025)	0/306
T.angkijvanich et al 1999	0.465 (0.371, 0.563)	47/101
Sulaiman and Sulaiman 1989	0.235 (0.184, 0.294)	53/226
Pramoolsins.ap et al 1994	0.633 (0.533, 0.722)	62/98
Tangkijvanich et al 2003	0.003 (0.000, 0.041)	0/188
Norsa'adah et al 2013	0.376 (0.313, 0.444)	79/210
Tangkijvanic..h et al 1999	0.002 (0.000, 0.038)	0/200
Makkoch et al 2016	0.001 (0.000, 0.021)	0/369
Omar et al 2015	0.001 (0.000, 0.022)	0/348
Sooklim et al 2003	0.003 (0.000, 0.043)	0/180
Somboon et al 2014	0.023 (0.011, 0.047)	7/308
Hoan et al 2019	0.122 (0.095, 0.156)	54/443
Chanthra et al 2016	0.001 (0.000, 0.014)	0/582
Sopipong et al 2013	0.001 (0.000, 0.020)	0/398
Poh et al 2015	0.082 (0.063, 0.105)	55/673
Chassagne et al 2016	0.054 (0.038, 0.077)	30/553
Lingao 1989	0.456 (0.404, 0.509)	155/340
Pawarode et al 2000	0.128 (0.097, 0.166)	47/368
Sriprapun et al 2016	0.572 (0.518, 0.625)	186/325
Liew et al 2019	0.000 (0.000, 0.007)	0/1079
Lim et al 2021	0.000 (0.000, 0.003)	0/3013
Nguyen-Dinh et al 2018	0.000 (0.000, 0.000)	0/24091
**Overall (I^2=96.96 % , P< 0.001)**	**0.041 (0.025, 0.068)**	**916/39050**

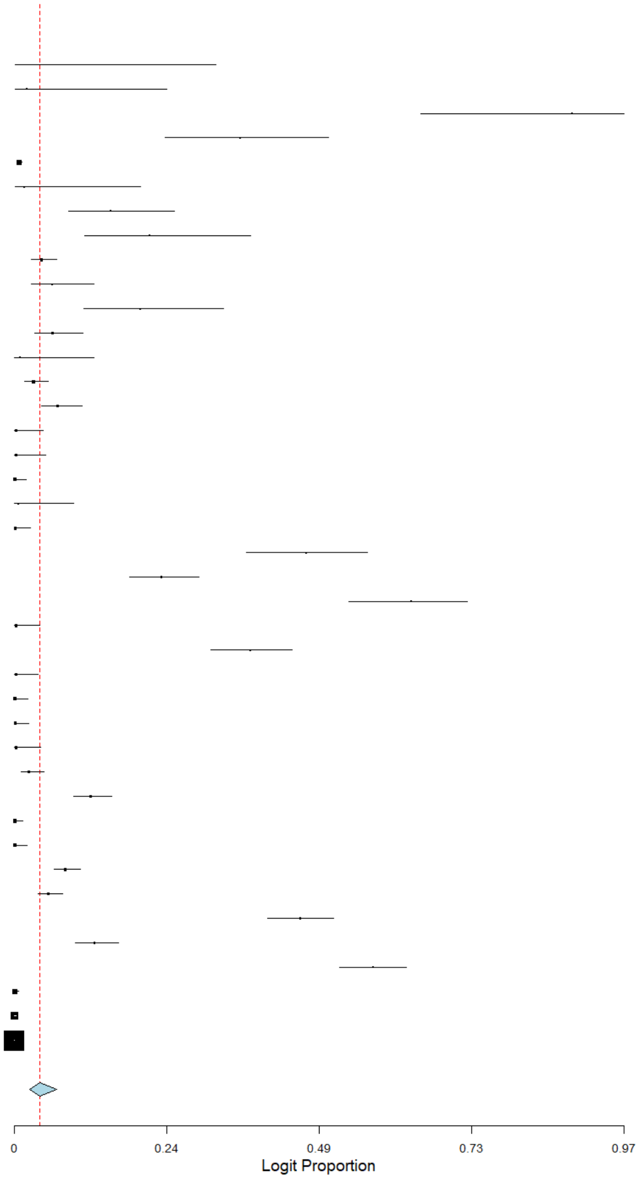

INTERMEDIATE

Studies	Estimate (95% C.I.)	Ev/Trt
Pongpipat et al 1983	0.028 (0.002, 0.322)	0/17
Doury et al 1978	0.019 (0.001, 0.244)	0/25
Chan et al 1980	0.026 (0.002, 0.310)	0/18
Nun-Anan et al 2015	0.120 (0.055, 0.242)	6/50
Kamalapirat et al 2021	0.004 (0.002, 0.008)	9/2208
Wungu et al 2018	0.015 (0.001, 0.201)	0/32
Tangkijvanich et al 2001	0.042 (0.014, 0.121)	3/72
Nørredam et al 1986	0.013 (0.001, 0.178)	0/37
Tongsiri et al 2017	0.013 (0.005, 0.030)	5/396
Sakamoto et al 2006	0.090 (0.047, 0.164)	9/100
Tan et al 1977	0.010 (0.001, 0.138)	0/50
Wanich et al 2016	0.037 (0.017, 0.079)	6/164
Khin et al 1996	0.009 (0.001, 0.127)	0/55
Jack et al 2019	0.055 (0.036, 0.084)	20/362
Huong et 2022	0.002 (0.000, 0.031)	0/247
Pramools.insap et al 1992	0.003 (0.000, 0.045)	0/168
Irie et al 1985	0.003 (0.000, 0.051)	0/150
Chao et al 1994	0.001 (0.000, 0.019)	0/423
Lim et al 1986	0.006 (0.000, 0.094)	0/77
Welsh et al 1976	0.002 (0.000, 0.025)	0/306
Tangk.ijvanich et al 1999	0.005 (0.000, 0.073)	0/101
Sulaiman and Sulaiman 1989	0.002 (0.000, 0.034)	0/226
Pr.amoolsinsap et al 1994	0.005 (0.000, 0.076)	0/98
Ta.ngkijvanich et al 2003	0.003 (0.000, 0.041)	0/188
Norsa'adah et al 2013	0.276 (0.220, 0.341)	58/210
Tangkijvanich. et al 1999	0.002 (0.000, 0.038)	0/200
Makkoch et al 2016	0.001 (0.000, 0.021)	0/369
Omar et al 2015	0.135 (0.103, 0.175)	47/348
Sooklim et al 2003	0.003 (0.000, 0.043)	0/180
Somboon et al 2014	0.159 (0.122, 0.204)	49/308
Hoan et al 2019	0.001 (0.000, 0.018)	0/443
Chanthra et al 2016	0.001 (0.000, 0.014)	0/582
Sopipong et al 2013	0.001 (0.000, 0.020)	0/398
Poh et al 2015	0.001 (0.000, 0.012)	0/673
Chassagne et al 2016	0.001 (0.000, 0.014)	0/553
Lingao 1989	0.001 (0.000, 0.023)	0/340
Pawarode et al 2000	0.001 (0.000, 0.021)	0/368
Sriprapun et al 2016	0.369 (0.318, 0.423)	120/325
Liew et al 2019	0.515 (0.485, 0.545)	556/1079
Lim et al 2021	0.000 (0.000, 0.003)	0/3013
Nguyen-Dinh et al 2018	0.000 (0.000, 0.000)	0/24091
**Overall (I^2=96.41 % , P< 0.001)**	**0.011 (0.006, 0.020)**	**888/39050**

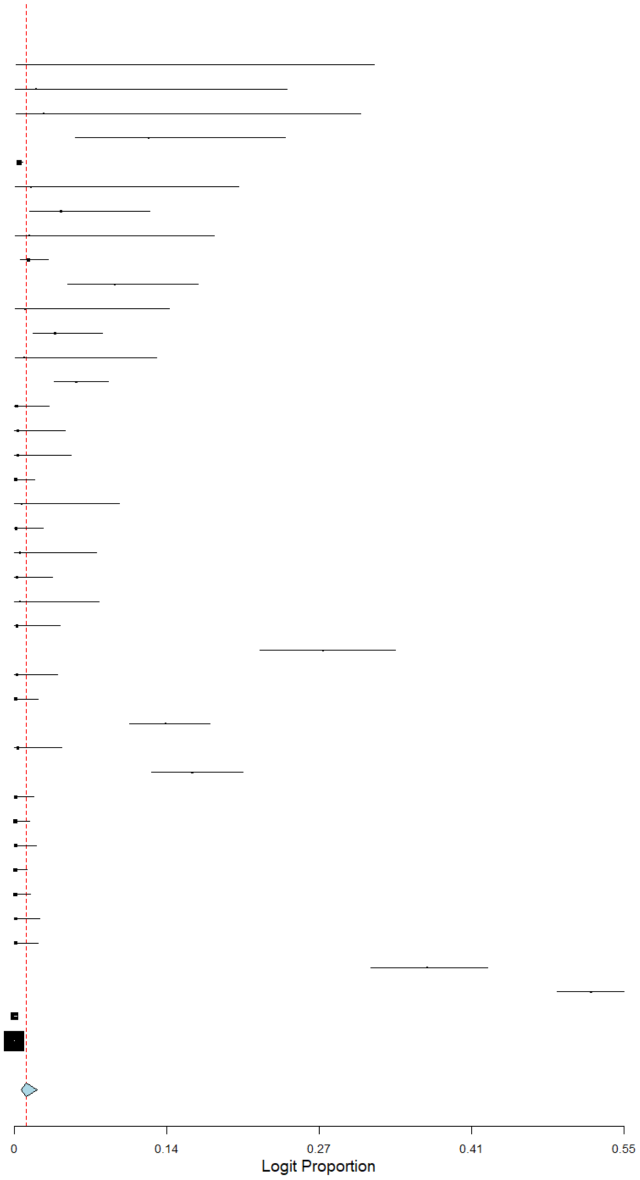

LATE

Studies	Estimate (95% C.I.)	Ev/Trt
Pongpipat et al 1983	0.028 (0.002, 0.322)	0/17
Doury et al 1978	0.019 (0.001, 0.244)	0/25
Chan et al 1980	0.026 (0.002, 0.310)	0/18
Nun-Anan et al 2015	0.040 (0.010, 0.146)	2/50
Kamalapirat et al 2021	0.002 (0.001, 0.005)	4/2208
Wungu et al 2018	0.015 (0.001, 0.201)	0/32
Tangkijv.anich et al 2001	0.042 (0.014, 0.121)	3/72
Nørredam et al 1986	0.013 (0.001, 0.178)	0/37
Tongsiri et al 2017	0.018 (0.008, 0.037)	7/396
Sakamoto et al 2006	0.100 (0.055, 0.176)	10/100
Tan et al 1977	0.010 (0.001, 0.138)	0/50
Wanich et al 2016	0.073 (0.042, 0.124)	12/164
Khin et al 1996	0.009 (0.001, 0.127)	0/55
Jack et al 2019	0.052 (0.034, 0.081)	19/362
Huong et 2022	0.002 (0.000, 0.031)	0/247
Pra.moolsinsap et al 1992	0.003 (0.000, 0.045)	0/168
Irie et al 1985	0.003 (0.000, 0.051)	0/150
Chao et al 1994	0.001 (0.000, 0.019)	0/423
Lim et al 1986	0.006 (0.000, 0.094)	0/77
Welsh et al 1976	0.002 (0.000, 0.025)	0/306
T.angkijvanich et al 1999	0.005 (0.000, 0.073)	0/101
Sulaiman and Sulaiman 1989	0.002 (0.000, 0.034)	0/226
Pramoolsinsa.p et al 1994	0.005 (0.000, 0.076)	0/98
Tangkijvanic.h et al 2003	0.003 (0.000, 0.041)	0/188
Norsa'adah et al 2013	0.119 (0.082, 0.170)	25/210
Tang.kijvanich et al 1999	0.002 (0.000, 0.038)	0/200
Makkoch et al 2016	0.001 (0.000, 0.021)	0/369
Omar et al 2015	0.126 (0.095, 0.166)	44/348
Sooklim et al 2003	0.003 (0.000, 0.043)	0/180
Somboon et al 2014	0.175 (0.137, 0.222)	54/308
Hoan et al 2019	0.001 (0.000, 0.018)	0/443
Chanthra et al 2016	0.001 (0.000, 0.014)	0/582
Sopipong et al 2013	0.001 (0.000, 0.020)	0/398
Poh et al 2015	0.001 (0.000, 0.012)	0/673
Chassagne et al 2016	0.001 (0.000, 0.014)	0/553
Lingao 1989	0.001 (0.000, 0.023)	0/340
Pawarode et al 2000	0.001 (0.000, 0.021)	0/368
Sriprapun et al 2016	0.160 (0.124, 0.204)	52/325
Liew et al 2019	0.151 (0.131, 0.174)	163/1079
Lim et al 2021	0.000 (0.000, 0.003)	0/3013
Nguyen-Dinh et al 2018	0.000 (0.000, 0.000)	0/24091
**Overall (I^2=90.18 % , P< 0.001)**	**0.013 (0.008, 0.020)**	**395/39050**

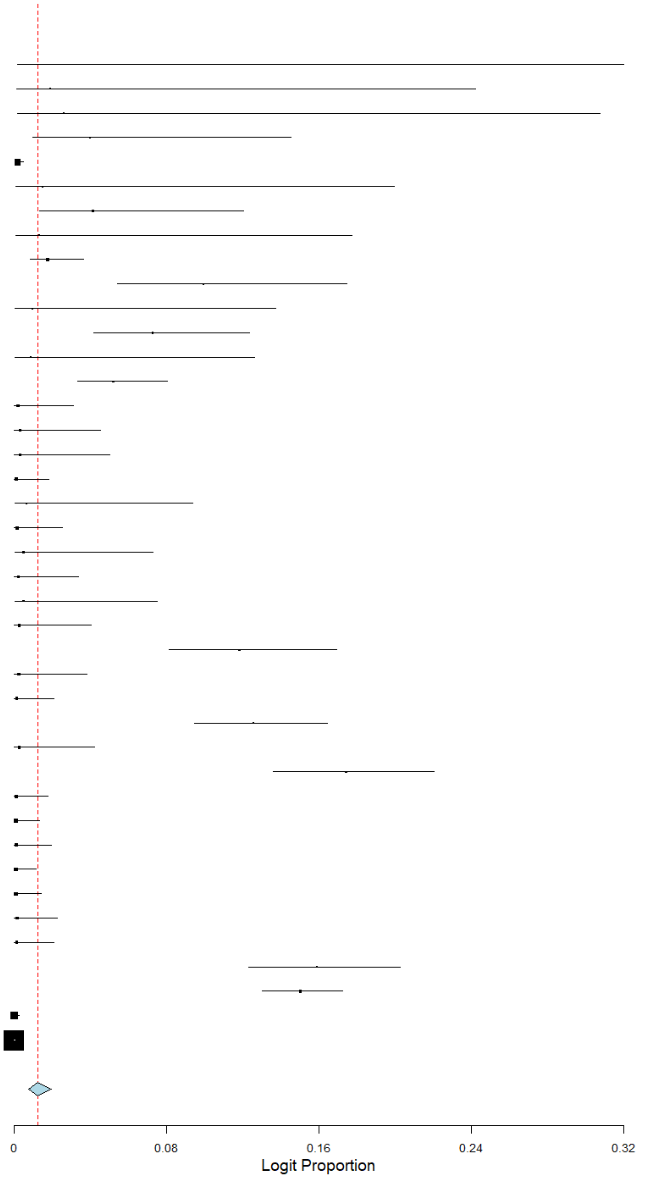

AFP YES

Studies	Estimate (95% C.I.)	Ev/Trt
Pongpipat et al 1983	0.471 (0.233, 0.708)	8/17
Doury et al 1978	0.019 (0.000, 0.072)	0/25
Chan et al 1980	0.611 (0.386, 0.836)	11/18
Nun-Anan et al 2015	0.010 (0.000, 0.037)	0/50
Kamalapirat et al 2021	0.000 (0.000, 0.001)	0/2208
Wungu et al 2018	0.015 (0.000, 0.057)	0/32
Tang.kijvanich et al 2001	0.007 (0.000, 0.026)	0/72
Nørredam et al 1986	0.013 (0.000, 0.049)	0/37
Tongsiri et al 2017	0.053 (0.031, 0.075)	21/396
Sakamoto et al 2006	0.005 (0.000, 0.019)	0/100
Tan et al 1977	0.520 (0.382, 0.658)	26/50
Wanich et al 2016	0.140 (0.087, 0.193)	23/164
Khin et al 1996	0.009 (0.000, 0.034)	0/55
Jack et al 2019	0.113 (0.081, 0.146)	41/362
Huong et 2022	0.002 (0.000, 0.008)	0/247
Pramoolsinsap et al 1992	0.003 (0.000, 0.011)	0/168
Irie et al 1985	0.003 (0.000, 0.012)	0/150
Chao et al 1994	0.001 (0.000, 0.004)	0/423
Lim et al 1986	0.792 (0.702, 0.883)	61/77
Welsh et al 1976	0.002 (0.000, 0.006)	0/306
T.angkijvanich et al 1999	0.005 (0.000, 0.018)	0/101
Sulaiman and Sulaiman 1989	0.002 (0.000, 0.008)	0/226
Pramoolsins.ap et al 1994	0.005 (0.000, 0.019)	0/98
Tangkijvanich et al 2003	0.003 (0.000, 0.010)	0/188
Norsa'adah et al 2013	0.576 (0.509, 0.643)	121/210
Tangkijvanic..h et al 1999	0.002 (0.000, 0.009)	0/200
Makkoch et al 2016	0.001 (0.000, 0.005)	0/369
Omar et al 2015	0.001 (0.000, 0.005)	0/348
Sooklim et al 2003	0.003 (0.000, 0.010)	0/180
Somboon et al 2014	0.002 (0.000, 0.006)	0/308
Hoan et al 2019	0.001 (0.000, 0.004)	0/443
Chanthra et al 2016	0.001 (0.000, 0.003)	0/582
Sopipong et al 2013	0.001 (0.000, 0.005)	0/398
Poh et al 2015	0.001 (0.000, 0.003)	0/673
Chassagne et al 2016	0.363 (0.323, 0.404)	201/553
Lingao 1989	0.732 (0.685, 0.779)	249/340
Pawarode et al 2000	0.001 (0.000, 0.005)	0/368
Sriprapun et al 2016	0.002 (0.000, 0.006)	0/325
Liew et al 2019	0.000 (0.000, 0.002)	0/1079
Lim et al 2021	0.000 (0.000, 0.001)	0/3013
Nguyen-Dinh et al 2018	0.000 (0.000, 0.000)	0/24091
**Overall (I^2=98.03 % , P< 0.001)**	**0.007 (0.005, 0.010)**	**762/39050**

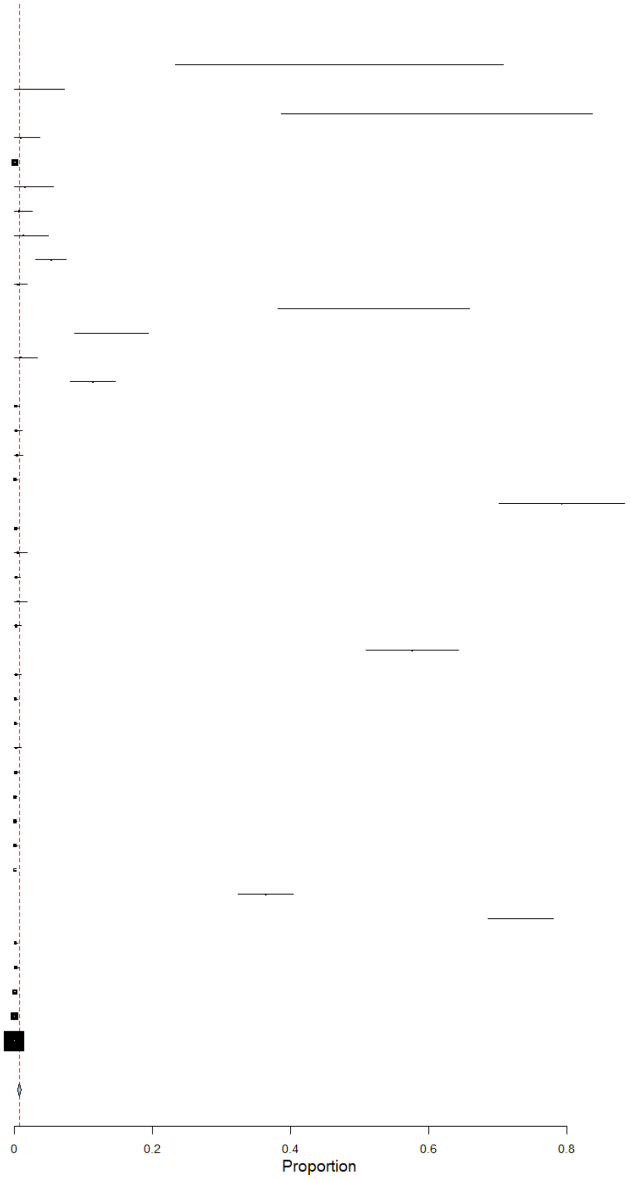

AFP NO

Studies	Estimate (95% C.I.)	Ev/Trt
Pongpipat et al 1983	0.294 (0.128, 0.542)	5/17
Doury et al 1978	0.019 (0.001, 0.244)	0/25
Chan et al 1980	0.444 (0.240, 0.670)	8/18
Nun-Anan et al 2015	0.010 (0.001, 0.138)	0/50
Kamalapirat et al 2021	0.000 (0.000, 0.004)	0/2208
Wungu et al 2018	0.015 (0.001, 0.201)	0/32
Tang.kijvanich et al 2001	0.007 (0.000, 0.100)	0/72
Nørredam et al 1986	0.013 (0.001, 0.178)	0/37
Tongsiri et al 2017	0.013 (0.005, 0.030)	5/396
Sakamoto et al 2006	0.005 (0.000, 0.074)	0/100
Tan et al 1977	0.220 (0.126, 0.355)	11/50
Wanich et al 2016	0.091 (0.056, 0.146)	15/164
Khin et al 1996	0.009 (0.001, 0.127)	0/55
Jack et al 2019	0.022 (0.011, 0.044)	8/362
Huong et 2022	0.002 (0.000, 0.031)	0/247
Pramoolsinsap et al 1992	0.003 (0.000, 0.045)	0/168
Irie et al 1985	0.003 (0.000, 0.051)	0/150
Chao et al 1994	0.001 (0.000, 0.019)	0/423
Lim et al 1986	0.208 (0.131, 0.313)	16/77
Welsh et al 1976	0.002 (0.000, 0.025)	0/306
T.angkijvanich et al 1999	0.005 (0.000, 0.073)	0/101
Sulaiman and Sulaiman 1989	0.002 (0.000, 0.034)	0/226
Pramoolsins.ap et al 1994	0.005 (0.000, 0.076)	0/98
Tangkijvanich et al 2003	0.003 (0.000, 0.041)	0/188
Norsa'adah et al 2013	0.002 (0.000, 0.037)	0/210
Tangkijvanic..h et al 1999	0.002 (0.000, 0.038)	0/200
Makkoch et al 2016	0.001 (0.000, 0.021)	0/369
Omar et al 2015	0.001 (0.000, 0.022)	0/348
Sooklim et al 2003	0.003 (0.000, 0.043)	0/180
Somboon et al 2014	0.002 (0.000, 0.025)	0/308
Hoan et al 2019	0.001 (0.000, 0.018)	0/443
Chanthra et al 2016	0.001 (0.000, 0.014)	0/582
Sopipong et al 2013	0.001 (0.000, 0.020)	0/398
Poh et al 2015	0.001 (0.000, 0.012)	0/673
Chassagne et al 2016	0.045 (0.031, 0.066)	25/553
Lingao 1989	0.015 (0.006, 0.035)	5/340
Pawarode et al 2000	0.001 (0.000, 0.021)	0/368
Sriprapun et al 2016	0.002 (0.000, 0.024)	0/325
Liew et al 2019	0.000 (0.000, 0.007)	0/1079
Lim et al 2021	0.000 (0.000, 0.003)	0/3013
Nguyen-Dinh et al 2018	0.000 (0.000, 0.000)	0/24091
**Overall (I^2=88.07 % , P< 0.001)**	**0.006 (0.003, 0.012)**	**98/39050**

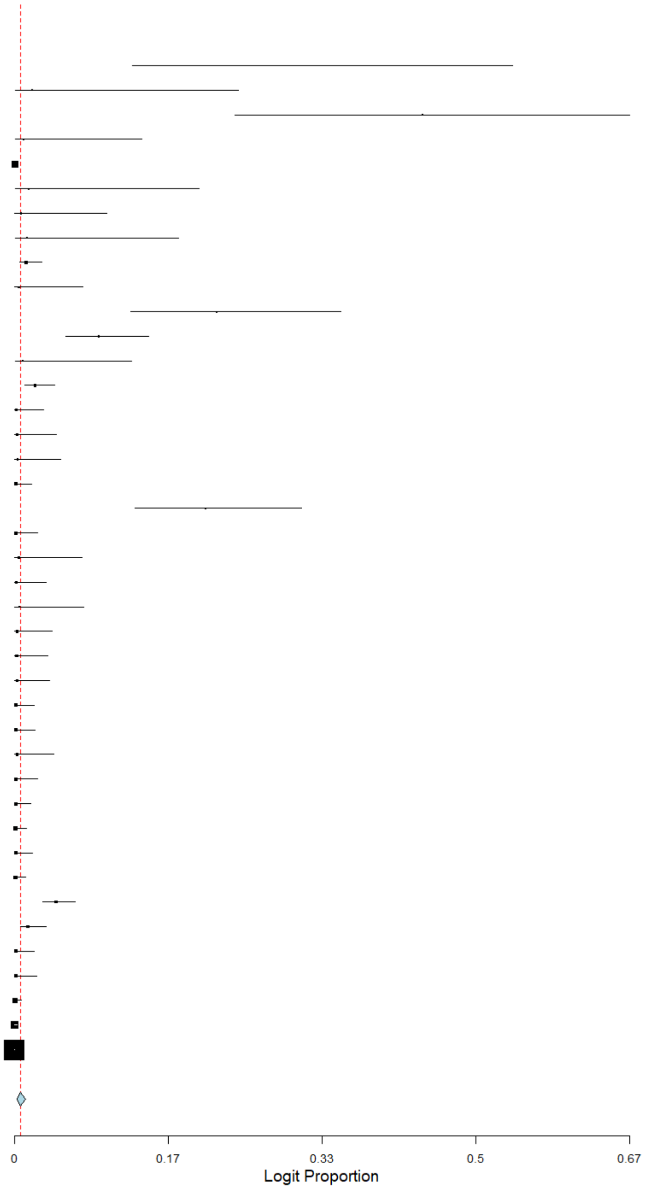

CIRHOSIS YES

Studies	Estimate (95% C.I.)	Ev/Trt
Pongpipat et al 1983	0.028 (0.002, 0.322)	0/17
Doury et al 1978	0.019 (0.001, 0.244)	0/25
Chan et al 1980	0.056 (0.008, 0.307)	1/18
Nun-Anan et al 2015	0.020 (0.003, 0.129)	1/50
Kamalapirat et al 2021	0.001 (0.000, 0.004)	3/2208
Wungu et al 2018	0.015 (0.001, 0.201)	0/32
Tang.kijvanich et al 2001	0.153 (0.087, 0.255)	11/72
Nørredam et al 1986	0.432 (0.284, 0.594)	16/37
Tongsiri et al 2017	0.023 (0.012, 0.043)	9/396
Sakamoto et al 2006	0.250 (0.175, 0.344)	25/100
Tan et al 1977	0.540 (0.402, 0.672)	27/50
Wanich et al 2016	0.171 (0.121, 0.236)	28/164
Khin et al 1996	0.009 (0.001, 0.127)	0/55
Jack et al 2019	0.105 (0.077, 0.141)	38/362
Huong et 2022	0.130 (0.093, 0.177)	32/247
Pramoolsinsap et al 1992	0.003 (0.000, 0.045)	0/168
Irie et al 1985	0.003 (0.000, 0.051)	0/150
Chao et al 1994	0.001 (0.000, 0.019)	0/423
Lim et al 1986	0.006 (0.000, 0.094)	0/77
Welsh et al 1976	0.002 (0.000, 0.025)	0/306
T.angkijvanich et al 1999	0.188 (0.123, 0.276)	19/101
Sulaiman and Sulaiman 1989	0.128 (0.091, 0.179)	29/226
Pramoolsins.ap et al 1994	0.214 (0.144, 0.306)	21/98
Tangkijvanich et al 2003	0.003 (0.000, 0.041)	0/188
Norsa'adah et al 2013	0.200 (0.151, 0.260)	42/210
Tangkijvanic..h et al 1999	0.002 (0.000, 0.038)	0/200
Makkoch et al 2016	0.001 (0.000, 0.021)	0/369
Omar et al 2015	0.001 (0.000, 0.022)	0/348
Sooklim et al 2003	0.003 (0.000, 0.043)	0/180
Somboon et al 2014	0.474 (0.419, 0.530)	146/308
Hoan et al 2019	0.264 (0.225, 0.307)	117/443
Chanthra et al 2016	0.001 (0.000, 0.014)	0/582
Sopipong et al 2013	0.001 (0.000, 0.020)	0/398
Poh et al 2015	0.229 (0.199, 0.262)	154/673
Chassagne et al 2016	0.354 (0.316, 0.395)	196/553
Lingao 1989	0.291 (0.245, 0.342)	99/340
Pawarode et al 2000	0.573 (0.522, 0.623)	211/368
Sriprapun et al 2016	0.246 (0.202, 0.296)	80/325
Liew et al 2019	0.000 (0.000, 0.007)	0/1079
Lim et al 2021	0.000 (0.000, 0.003)	0/3013
Nguyen-Dinh et al 2018	0.000 (0.000, 0.000)	0/24091
**Overall (I^2=95.17 % , P< 0.001)**	**0.067 (0.047, 0.095)**	**1305/39050**

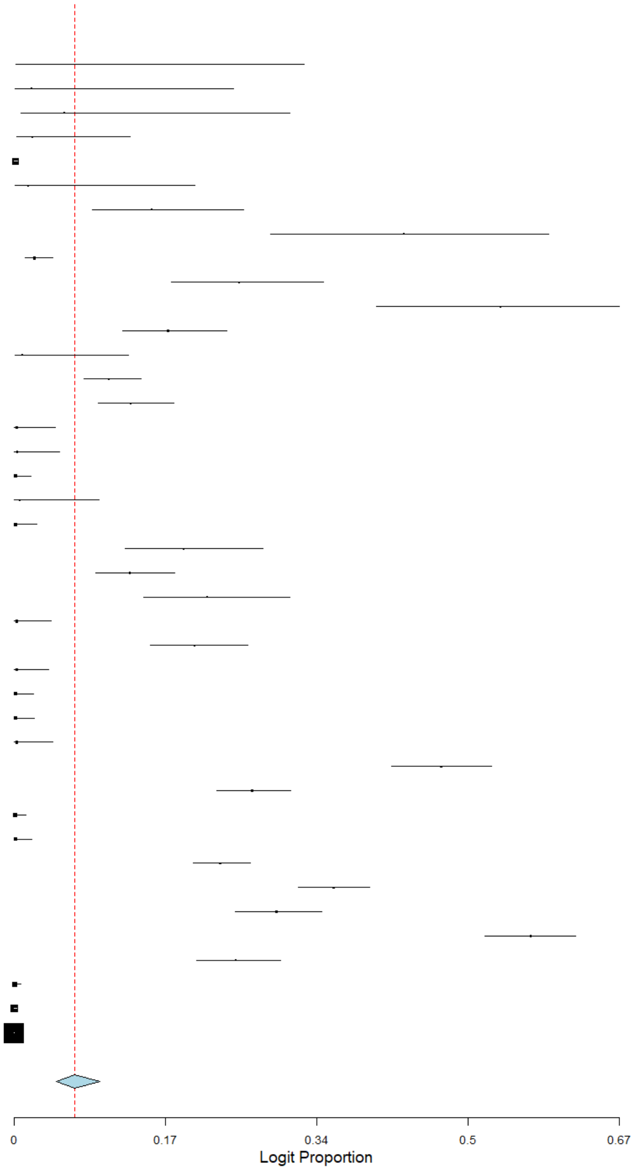

CIRRHOSIS NO

Studies	Estimate (95% C.I.)	Ev/Trt
Pongpipat et al 1983	0.028 (0.002, 0.322)	0/17
Doury et al 1978	0.019 (0.001, 0.244)	0/25
Chan et al 1980	0.889 (0.648, 0.972)	16/18
Nun-Anan et al 2015	0.360 (0.240, 0.501)	18/50
Kamalapirat et al 2021	0.008 (0.005, 0.012)	17/2208
Wungu et al 2018	0.015 (0.001, 0.201)	0/32
Tangkijvanich et al 2001	0.153 (0.087, 0.255)	11/72
Nørredam et al 1986	0.216 (0.112, 0.376)	8/37
Tongsiri et al 2017	0.043 (0.027, 0.068)	17/396
Sakamoto et al 2006	0.060 (0.027, 0.127)	6/100
Tan et al 1977	0.200 (0.111, 0.333)	10/50
Wanich et al 2016	0.061 (0.033, 0.110)	10/164
Khin et al 1996	0.009 (0.001, 0.127)	0/55
Jack et al 2019	0.030 (0.017, 0.054)	11/362
Huong et 2022	0.069 (0.043, 0.108)	17/247
Pramools.insap et al 1992	0.003 (0.000, 0.045)	0/168
Irie et al 1985	0.003 (0.000, 0.051)	0/150
Chao et al 1994	0.001 (0.000, 0.019)	0/423
Lim et al 1986	0.006 (0.000, 0.094)	0/77
Welsh et al 1976	0.002 (0.000, 0.025)	0/306
Tangk.ijvanich et al 1999	0.465 (0.371, 0.563)	47/101
Sulaiman and Sulaiman 1989	0.235 (0.184, 0.294)	53/226
Pr.amoolsinsap et al 1994	0.633 (0.533, 0.722)	62/98
Ta.ngkijvanich et al 2003	0.003 (0.000, 0.041)	0/188
Norsa'adah et al 2013	0.376 (0.313, 0.444)	79/210
Tangkijvanich. et al 1999	0.002 (0.000, 0.038)	0/200
Makkoch et al 2016	0.001 (0.000, 0.021)	0/369
Omar et al 2015	0.001 (0.000, 0.022)	0/348
Sooklim et al 2003	0.003 (0.000, 0.043)	0/180
Somboon et al 2014	0.023 (0.011, 0.047)	7/308
Hoan et al 2019	0.122 (0.095, 0.156)	54/443
Chanthra et al 2016	0.001 (0.000, 0.014)	0/582
Sopipong et al 2013	0.001 (0.000, 0.020)	0/398
Poh et al 2015	0.082 (0.063, 0.105)	55/673
Chassagne et al 2016	0.054 (0.038, 0.077)	30/553
Lingao 1989	0.456 (0.404, 0.509)	155/340
Pawarode et al 2000	0.128 (0.097, 0.166)	47/368
Sriprapun et al 2016	0.572 (0.518, 0.625)	186/325
Liew et al 2019	0.000 (0.000, 0.007)	0/1079
Lim et al 2021	0.000 (0.000, 0.003)	0/3013
Nguyen-Dinh et al 2018	0.000 (0.000, 0.000)	0/24091
**Overall (I^2=96.96 % , P< 0.001)**	**0.041 (0.025, 0.068)**	**916/39050**

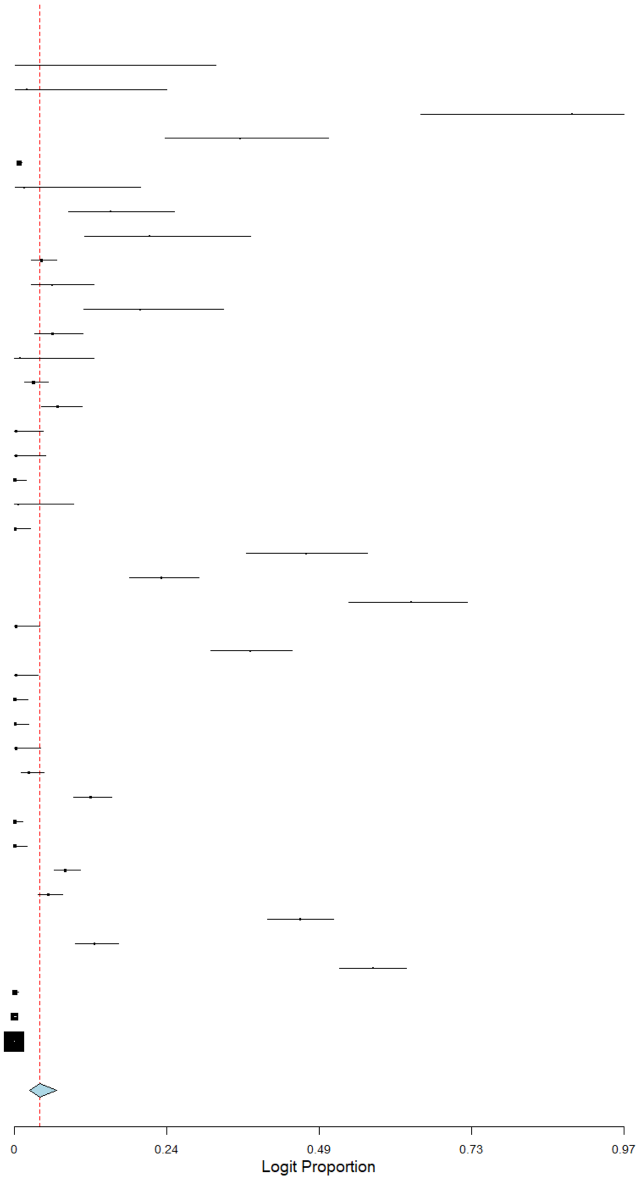

STUDY DESIGN

Studies	Estimate (95% C.I.)	Ev/Trt
Pongpipat et al., 1983	0.765 (0.514, 0.909)	13/17
Doury et al, 1978	0.640 (0.440, 0.801)	16/25
Chan et al., 1980	0.944 (0.693, 0.992)	17/18
Nun-Anan et al., 2015	0.380 (0.257, 0.520)	19/50
Kamalapirat et al., 2021	0.009 (0.006, 0.014)	20/2208
Wungu et al., 2018	0.656 (0.479, 0.798)	21/32
Tangkijvanich et al., 2001	0.306 (0.210, 0.421)	22/72
Nørredam et al., 1986	0.649 (0.485, 0.784)	24/37
Sakamoto et al., 2006	0.310 (0.227, 0.407)	31/100
Tan et al., 1977	0.740 (0.602, 0.843)	37/50
Wanich et al., 2016	0.232 (0.173, 0.302)	38/164
Lim et al., 1986	0.792 (0.687, 0.869)	61/77
Welsh et al., 1976	0.199 (0.158, 0.248)	61/306
Tangkijvanich et al., 1999	0.653 (0.556, 0.740)	66/101
Pramoolsinsap et al., 1994	0.847 (0.761, 0.906)	83/98
Norsa'adah et al., 2013	0.576 (0.508, 0.641)	121/210
Tangkijvanich et al., 1999	0.650 (0.581, 0.713)	130/200
Makkoch et al., 2016	0.382 (0.334, 0.433)	141/369
Omar et al., 2015	0.411 (0.360, 0.463)	143/348
Lingao, 1989	0.747 (0.698, 0.790)	254/340
Sriprapun et al., 2016	0.818 (0.773, 0.857)	266/325
**Subgroup Crossectional (I^2=97.86 % , P=0.000)**	**0.530 (0.391, 0.665)**	**1584/5147**
Tongsiri et al, 2017	0.066 (0.045, 0.095)	26/396
Jack et al., 2019	0.135 (0.104, 0.175)	49/362
Huong et., 2022	0.198 (0.153, 0.253)	49/247
Irie et al., 1985	0.373 (0.300, 0.453)	56/150
Chao et al., 1994	0.137 (0.108, 0.173)	58/423
Sulaiman and Sulaiman, 1989	0.363 (0.303, 0.428)	82/226
Tangkijvanich et al., 2003	0.559 (0.487, 0.628)	105/188
Sooklim et al., 2003	0.817 (0.753, 0.867)	147/180
Somboon et al., 2014	0.497 (0.441, 0.552)	153/308
Chassagne et al., 2016	0.409 (0.368, 0.450)	226/553
Pawarode et al., 2000	0.701 (0.652, 0.746)	258/368
Liew et al., 2019	0.849 (0.826, 0.869)	916/1079
Lim et al., 2021	0.466 (0.448, 0.484)	1404/3013
Nguyen-Dinh et al., 2018	0.062 (0.059, 0.065)	1501/24091
**Subgroup retrospective (I^2=99.79 % , P=0.000)**	**0.365 (0.187, 0.590)**	**5030/31584**
Khin et al., 1996	0.745 (0.615, 0.843)	41/55
Pramoolsinsap et al., 1992	0.298 (0.233, 0.371)	50/168
Hoan et al., 2019	0.386 (0.342, 0.432)	171/443
Chanthra et al., 2016	0.330 (0.293, 0.369)	192/582
Sopipong et al., 2013	0.508 (0.459, 0.556)	202/398
Poh et al., 2015	0.311 (0.277, 0.347)	209/673
**Subgroup Case control (I^2=93.48 % , P=0.000)**	**0.412 (0.329, 0.500)**	**865/2319**
**Overall (I^2=99.51 % , P=0.000)**	**0.458 (0.343, 0.578)**	**7479/39050**

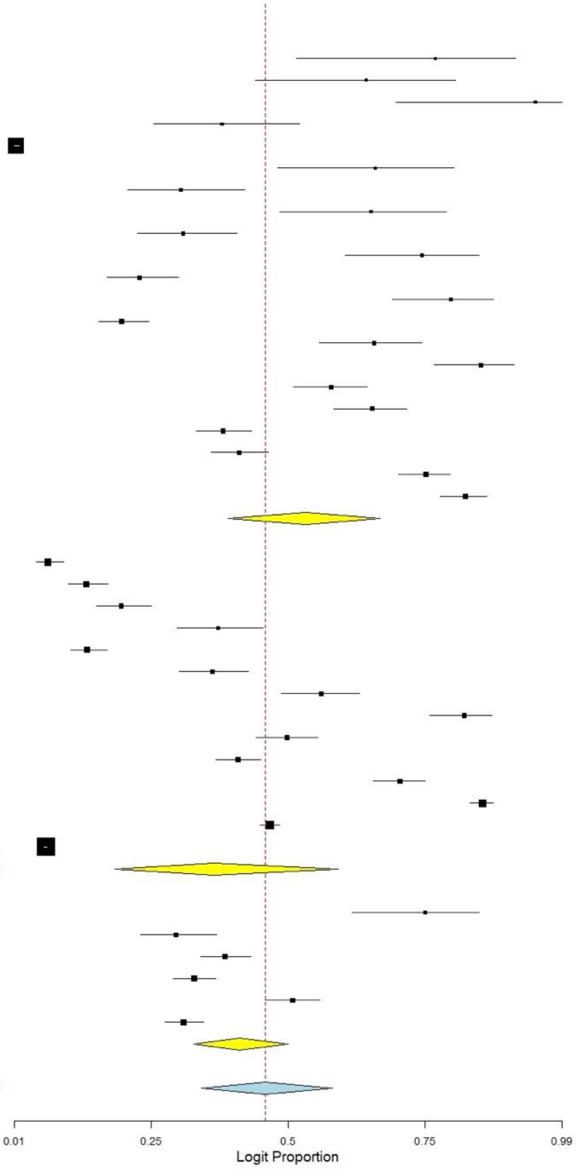

YEAR OF STUDY

Studies	Estimate (95% C.I.)	Ev/Trt
Welsh et al., 1976	0.199 (0.158, 0.248)	61/306
Tan et al., 1977	0.740 (0.602, 0.843)	37/50
Doury et al, 1978	0.640 (0.440, 0.801)	16/25
Pramoolsinsap et al., 1992	0.298 (0.233, 0.371)	50/168
Tangkijvanich et al., 1999	0.650 (0.581, 0.713)	130/200
Pawarode et al., 2000	0.701 (0.652, 0.746)	258/368
Sooklim et al., 2003	0.817 (0.753, 0.867)	147/180
**Subgroup 1975 - 1985 (I^2=97.66 % , P=0.000)**	**0.580 (0.371, 0.764)**	**699/1297**
Chan et al., 1980	0.944 (0.693, 0.992)	17/18
Lingao, 1989	0.747 (0.698, 0.790)	254/340
Nun-Anan et al., 2015	0.380 (0.257, 0.520)	19/50
Wanich et al., 2016	0.232 (0.173, 0.302)	38/164
Chanthra et al., 2016	0.330 (0.293, 0.369)	192/582
Tongsiri et al, 2017	0.066 (0.045, 0.095)	26/396
Wungu et al., 2018	0.656 (0.479, 0.798)	21/32
**Subgroup 2006 - 2015 (I^2=98.08 % , P=0.000)**	**0.441 (0.224, 0.682)**	**567/1582**
Pongpipat et al., 1983	0.765 (0.514, 0.909)	13/17
Irie et al., 1985	0.373 (0.300, 0.453)	56/150
Sulaiman and Sulaiman, 1989	0.363 (0.303, 0.428)	82/226
Chao et al., 1994	0.137 (0.108, 0.173)	58/423
Khin et al., 1996	0.745 (0.615, 0.843)	41/55
Tangkijvanich et al., 1999a	0.653 (0.556, 0.740)	66/101
Poh et al, 2015	0.311 (0.277, 0.347)	209/673
Chassagne et al., 2016	0.409 (0.368, 0.450)	226/553
Sriprapun et al., 2016	0.818 (0.773, 0.857)	266/325
Nguyen-Dinh et al., 2018	0.062 (0.059, 0.065)	1501/24091
Liew et al, 2019	0.849 (0.826, 0.869)	916/1079
Kamalapirat et al., 2021	0.009 (0.006, 0.014)	20/2208
Lim et al, 2021	0.466 (0.448, 0.484)	1404/3013
Huong et., 2022	0.198 (0.153, 0.253)	49/247
**Subgroup 2016 - 2023 (I^2=99.78 % , P=0.000)**	**0.376 (0.192, 0.605)**	**4907/33161**
Nørredam et al., 1986	0.649 (0.485, 0.784)	24/37
Pramoolsinsap et al., 1994	0.847 (0.761, 0.906)	83/98
Sakamoto et al., 2006	0.310 (0.227, 0.407)	31/100
Somboon et al, 2014	0.497 (0.441, 0.552)	153/308
Omar et al., 2015	0.411 (0.360, 0.463)	143/348
Makkoch et al., 2016	0.382 (0.334, 0.433)	141/369
Hoan et al., 2019	0.386 (0.342, 0.432)	171/443
**Subgroup 1996 - 2005 (I^2=92.1 % , P=0.000)**	**0.489 (0.397, 0.583)**	**746/1703**
Lim et al., 1986	0.792 (0.687, 0.869)	61/77
Tangkijvanich et al., 2001	0.306 (0.210, 0.421)	22/72
Tangkijvanich et al., 2003	0.559 (0.487, 0.628)	105/188
Norsa'adah et al., 2013	0.576 (0.508, 0.641)	121/210
Sopipong et al., 2013	0.508 (0.459, 0.556)	202/398
Jack et al., 2019	0.150 (0.115, 0.193)	49/326
**Subgroup 1986 - 1995 (I^2=96.87 % , P=0.000)**	**0.471 (0.304, 0.645)**	**560/1271**
**Overall (I^2=99.51 % , P=0.000)**	**0.459 (0.344, 0.579)**	**7479/39014**

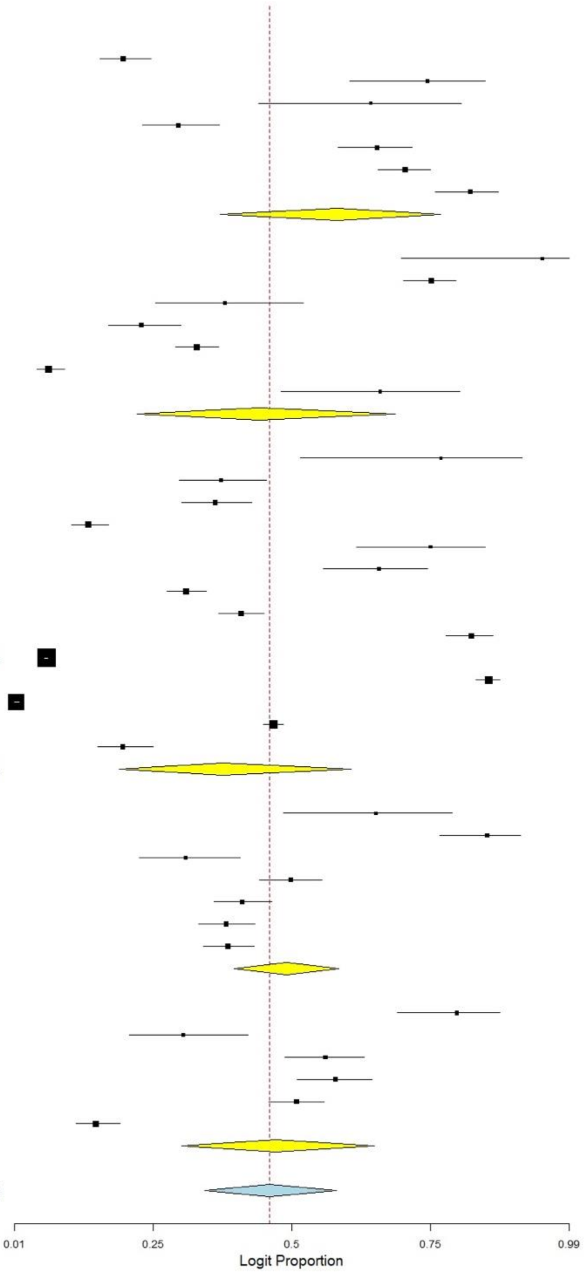

Supplement: Supplementary file 1 [file pathogens-12-01220-s001.zip › File S4_SUBGROUP META ANALYSIS HCC FILE_Revised.pdf]
