# Supplementary material for: Prevalence of Hepatocellular Carcinoma in Hepatitis B Population within Southeast Asia: A Systematic Review and Meta-Analysis of 39,050 Participants
Source: Pathogens. 2023 Oct 6;12(10):1220. doi: 10.3390/pathogens12101220 (PMC10609743; doi:10.3390/pathogens12101220)
Supplement: Supplementary file 1 [file pathogens-12-01220-s001.zip › Table S1_QUALITY OF INCLUDED STUDIES BY JBI CRITICAL APPRAISAL CHECKLIST FOR STUDIES REPORTING PREVALENCE DATA.pdf]

QUALITY OF INCLUDED STUDIES BY JBI CRITICAL APPRAISAL CHECKLIST FOR STUDIES REPORTING PREVALENCE DATA

S/N	Name of authors and year of publication		JBI checklist*									Total
1	Welsh et al., 1976	1976	1	2	3	4	5	6	7	8	9	
2	Tan et al., 1977	1977	Yes	No	Yes	Yes	Yes	Yes	Yes	Yes	Yes	16
3	Doury et al, 1978	1978	Yes	No	Yes	Yes	Yes	Yes	Yes	Yes	Yes	16
4	Chan et al., 1980	1980	Yes	No	Yes	Yes	Yes	Yes	Yes	Yes	Yes	16
5	Pongpipat et al., 1983	1983	Yes	No	Yes	Yes	Yes	Yes	Yes	Yes	Yes	16
6	Irie et al., 1985	1985	Yes	No	Yes	Yes	Yes	Yes	Yes	Yes	Yes	16
7	Nørredam et al., 1986	1986	Yes	No	Yes	Yes	Yes	Yes	Yes	Yes	Yes	16
8	Lim et al., 1986	1986	Yes	No	Yes	Yes	Yes	Yes	Yes	Yes	Yes	16
9	Sulaiman and Sulaiman, 1989	1989	Yes	No	Yes	Yes	Yes	Yes	Yes	Yes	Yes	16
10	Lingao, 1989	1989	Yes	No	Yes	Yes	Yes	Yes	Yes	Yes	Yes	16
11	Pramoolsinsap et al., 1992	1992	Yes	No	Yes	Yes	Yes	Yes	Yes	Yes	Yes	16
12	Chao et al., 1994	1994	Yes	No	Yes	Yes	Yes	Yes	Yes	Yes	Yes	16
13	Pramoolsinsap et al., 1994	1994	Yes	No	Yes	Yes	Yes	Yes	Yes	Yes	Yes	16
14	Khin et al., 1996	1996	Yes	No	Yes	Yes	Yes	Yes	Yes	Yes	Yes	16
15	Tangkijvanich et al., 1999a	1999	Yes	No	Yes	Yes	Yes	Yes	Yes	Yes	Yes	16
16	Tangkijvanich et al., 1999	1999	Yes	No	Yes	Yes	Yes	Yes	Yes	Yes	Yes	16
17	Pawarode et al., 2000	2000	Yes	No	Yes	Yes	Yes	Yes	Yes	Yes	Yes	16
18	Tangkijvanich et al., 2001	2001	Yes	No	Yes	Yes	Yes	Yes	Yes	Yes	Yes	16
19	Tangkijvanich et al., 2003	2003	Yes	No	Yes	Yes	Yes	Yes	Yes	Yes	Yes	16
20	Sooklim et al., 2003	2003	Yes	No	Yes	Yes	Yes	Yes	Yes	Yes	Yes	16
21	Sakamoto et al., 2006	2006	Yes	No	Yes	Yes	Yes	Yes	Yes	Yes	Yes	16
22	Norsa'adah et al., 2013	2013	Yes	No	Yes	Yes	Yes	Yes	Yes	Yes	Yes	16
23	Sopipong et al., 2013	2013	Yes	No	Yes	Yes	Yes	Yes	Yes	Yes	Yes	16
24	Somboon et al, 2014	2014	Yes	No	Yes	Yes	Yes	Yes	Yes	Yes	Yes	16
25	Nun-Anan et al., 2015	2015	Yes	No	Yes	Yes	Yes	Yes	Yes	Yes	Yes	16
26	Omar et al., 2015	2015	Yes	No	Yes	Yes	Yes	Yes	Yes	Yes	Yes	16
27	Poh et al, 2015	2015	Yes	Yes	Yes	Yes	Yes	Yes	Yes	Yes	Yes	18
28	Wanich et al., 2016	2016	Yes	Yes	Yes	Yes	Yes	Yes	Yes	Yes	Yes	18
29	Makkoch et al., 2016	2016	Yes	No	Yes	Yes	Yes	Yes	Yes	Yes	Yes	16
30	Chanthra et al., 2016	2016	Yes	No	Yes	Yes	Yes	Yes	Yes	Yes	Yes	16
31	Chassagne et al., 2016	2016	Yes	No	Yes	Yes	Yes	Yes	Yes	Yes	Yes	16
32	Sriprapun et al., 2016	2016	Yes	No	Yes	Yes	Yes	Yes	Yes	Yes	Yes	16
33	Tongsiri et al, 2017	2017	Yes	No	Yes	Yes	Yes	Yes	Yes	Yes	Yes	16
34	Wungu et al., 2018	2018	Yes	No	Yes	Yes	Yes	Yes	Yes	Yes	Yes	16
35	Nguyen-Dinh et al., 2018	2018	Yes	No	Yes	Yes	Yes	Yes	Yes	Yes	Yes	16
36	Jack et al., 2019	2019	Yes	No	Yes	Yes	Yes	Yes	Yes	Yes	Yes	16
37	Hoan et al., 2019	2019	Yes	Yes	Yes	Yes	Yes	Yes	Yes	Yes	Yes	18
38	Liew et al, 2019	2019	Yes	No	Yes	Yes	Yes	Yes	Yes	Yes	Yes	16
39	Kamalapirat et al., 2021	2021	Yes	Yes	Yes	Yes	Yes	Yes	Yes	Yes	Yes	18
40	Lim et al, 2021	2021	Yes	No	Yes	Yes	Yes	Yes	Yes	Yes	Yes	16
41	Huong et., 2022	2022	Yes	Yes	Yes	Yes	Yes	Yes	Yes	Yes	Yes	18

JBI CHECKLIST* 1. Appropriate sampling frame to address target population, **2.** Appropriate sampling way of study participants, **3.** Adequate sample size, **4.** Detail description of study participants and settings, **5.** Data analysis with sufficient coverage of identified sample, **6.** Use of valid methods to identify the condition, **7.** Standard, reliable way of measurement of condition for all participants, **8.** Availability of appropriate statistical analysis, **9.** Adequate response rate and management of low response rate.

Scores are coded as Yes=2 and No=0.
